# Otic Organoids Containing Spiral Ganglion Neuron-like Cells Derived from Human-induced Pluripotent Stem Cells as a Model of Drug-induced Neuropathy

**DOI:** 10.1093/stcltm/szab023

**Published:** 2022-03-07

**Authors:** Sho Kurihara, Masato Fujioka, Motoki Hirabayashi, Tomohiko Yoshida, Makoto Hosoya, Masashi Nagase, Fusao Kato, Kaoru Ogawa, Hideyuki Okano, Hiromi Kojima, Hirotaka James Okano

**Affiliations:** 1 Division of Regenerative Medicine, The Jikei University School of Medicine, 3-25-8 Nishishimbashi Minato-ku, Tokyo, Japan; 2 Department of Otorhinolaryngology, The Jikei University School of Medicine, 3-25-8 Nishishimbashi Minato-ku, Tokyo, Japan; 3 Department of Otorhinolaryngology, Head and Neck Surgery, Keio University School of Medicine, 35 Shinanomachi Shinjuku-ku, Tokyo, Japan; 4 Department of Neuroscience, The Jikei University School of Medicine, 3-25-8 Nishishimbashi Minato-ku, Tokyo, Japan; 5 Department of Physiology, Keio University School of Medicine, 35 Shinanomachi Shinjuku-ku, Tokyo, Japan

**Keywords:** inner ear, spiral ganglion, organoid, induced pluripotent stem cells, drug screening

## Abstract

The spiral ganglion of the cochlea is essential for hearing and contains primary bipolar neurons that relay action potentials generated by mechanosensory hair cells. Injury to spiral ganglion neurons (SGNs) causes permanent hearing loss because these cells have limited regenerative capacity. Establishment of human cell-derived inner ear tissue in vitro could facilitate the development of treatments for hearing loss. Here, we report a stepwise protocol for differentiating human-induced pluripotent stem cells (hiPSCs) into otic organoids that contain SGN-like cells and demonstrate that otic organoids have potential for use as an experimental model of drug-induced neuropathy. Otic progenitor cells (OPCs) were created by 2D culture of hiPSCs for 9 days. Otic spheroids were formed after 2D culture of OPCs for 2 days in a hypoxic environment. Otic organoids were generated by 3D culture of otic spheroids under hypoxic conditions for 5 days and normoxic conditions for a further 30 days or more. The protein expression profile, morphological characteristics, and electrophysiological properties of SGN-like cells in otic organoids were similar to those of primary SGNs. Live-cell imaging of AAV-syn-EGFP-labeled neurons demonstrated temporal changes in cell morphology and revealed the toxic effects of ouabain (which causes SGN-specific damage in animal experiments) and cisplatin (a chemotherapeutic drug with ototoxic adverse effects). Furthermore, a cyclin-dependent kinase-2 inhibitor suppressed the toxic actions of cisplatin on SGN-like cells in otic organoids. The otic organoid described here is a candidate novel drug screening system and could be used to identify drugs for the prevention of cisplatin-induced neuropathy.

## Graphical Abstract

**Figure:**
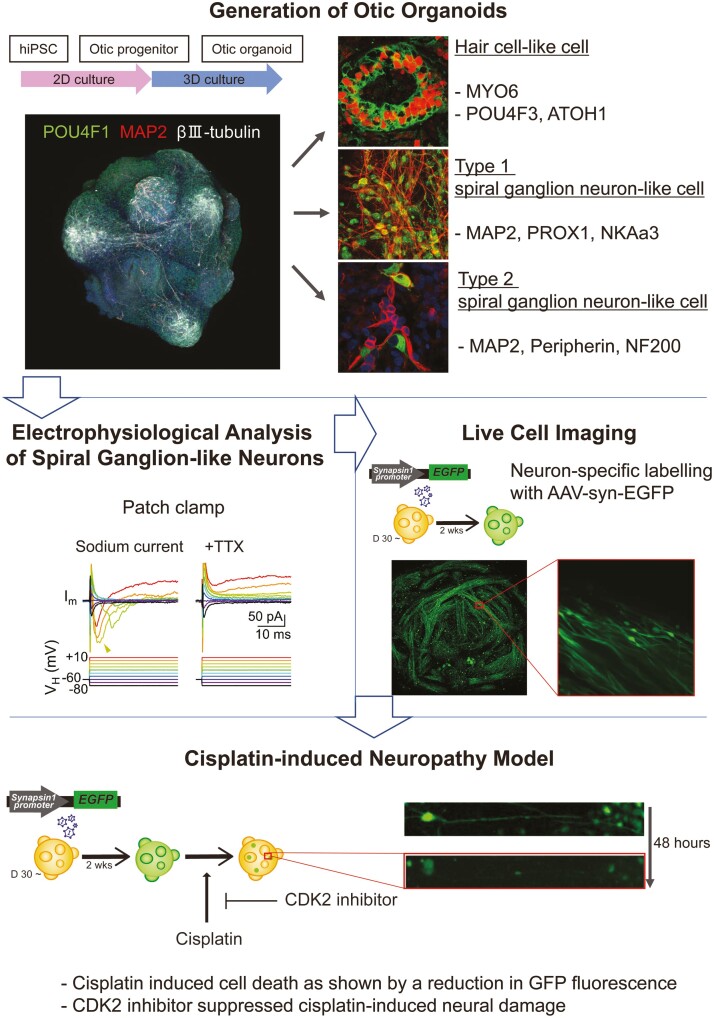
We report a protocol for differentiating human-induced pluripotent stem cells to otic organoids based on 2D and 3D culture techniques. Ototoxic effects of cisplatin and otoprotective effects of a CDK2 inhibitor were investigated in spiral ganglion neuron-like cells on the organoid surface. Otic organoids could be used to facilitate pathophysiological analyses and the development of treatments for hearing loss.

**Significance Statement**

In this study, we developed a highly efficient protocol to induce a uniform population of otic progenitor cells in two-dimensional culture, before shifting to three-dimensional culture to promote autonomous differentiation and maturation. On the surface of otic organoids, neuronal cells with gene expression patterns similar to those of native spiral ganglion neurons were identified. These cells showed neuron-specific voltage- and time-dependent currents and exhibited two distinct firing patterns similar to those seen in rodent spiral ganglion neurons. Our experimental approach, which involves electrophysiological analysis and live-cell imaging, will be useful for the development of a treatment for hearing loss.

## Introduction

Mammalian cochlear hair cells convert audible sounds (oscillations in air pressure) into electrical signals that are transmitted to the cochlear nucleus by spiral ganglion neurons (SGNs). Hair cells and SGNs can be affected by acoustic trauma, genetic diseases, and ototoxic drugs, and their death leads to permanent sensorineural hearing loss because they cannot be regenerated. This makes the treatment of sensorineural hearing loss highly challenging, and currently, there are no curative therapies.

The development of new treatments for sensorineural hearing loss requires suitable model systems. Although animal experiments provide useful insights, mouse models may not fully recapitulate the phenotype of hearing loss in humans.^1,2^ The limitations of non-human model systems potentially can be overcome using stem cell technology to generate human inner ear cells in vitro. Furthermore, the robust proliferation of stem cells enables their use in high-throughput drug screening assays.

Protocols for producing inner ear cells from human stem cells have been reported previously. Two-dimensional (2D) multistep culture was used to induce the differentiation of human embryonic stem cells (hESCs) into inner ear progenitor cells that developed into hair cell-like cells and auditory neurons.^3^ Another stepwise procedure was combined with cell sorting to generate SGN-like cells from hESCs.^4^ Furthermore, a three-dimensional (3D) culture technique was used to engineer mature hair cell-like cells and sensory neurons from hESCs and human-induced pluripotent stem cells (hiPSCs).^5^ Previous studies have also described protocols for producing sensory neurons from other human stem cells with a view to developing SGN replacement therapies.^6-8^ Recently, it has been reported that iPSC-derived SGNs could be formed in the mouse fetus using blastocyst complementation.^9^ hiPSCs are a readily available source of human stem cells with multilineage potential, and cells differentiated from patient-derived iPSCs are particularly well suited to genetic disease modeling.^10^ Furthermore, hiPSC-derived tissues potentially could be used as experimental systems for drug screening, elucidation of drug mechanisms, and development of novel therapies.

**Figure 1.**
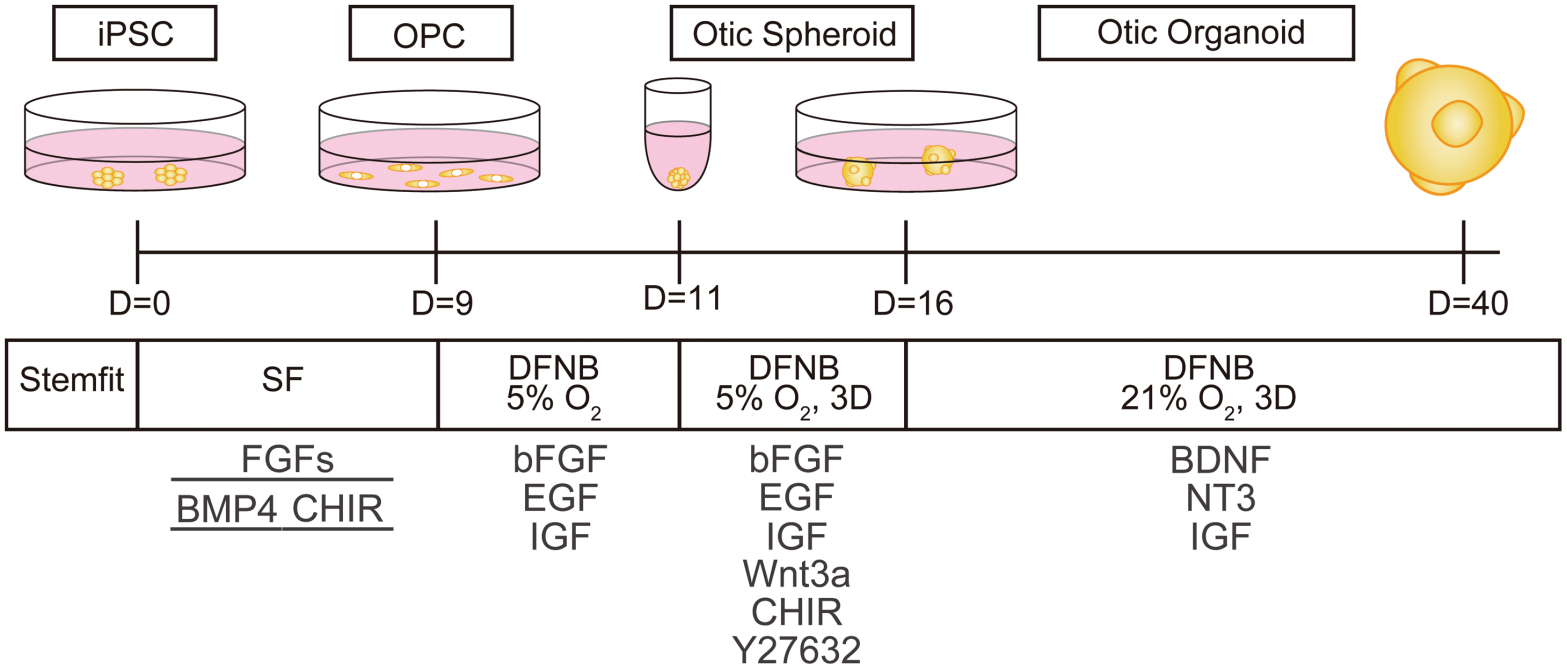
Schematic diagram showing the generation of otic organoids from hiPSCs. OPCs were generated by the culture of hiPSCs under 2D conditions for 9 days. Otic spheroids were formed by culturing OPCs under 2D conditions for 2 days (days 9-11) in a hypoxic environment. Otic spheroids were then cultured under 3D conditions for 5 days (days 11-16) in a hypoxic environment so that they formed solid tissues. Subsequent culture of otic spheroids under 3D conditions in a normoxic environment generated otic organoids. Abbreviations: hiPSCs, human-induced pluripotent stem cells; OPCs, otic progenitor cells.

Here, we describe a novel, high-efficiency protocol for generating hiPSC-derived otic organoids containing SGN-like cells and hair cell-like cells. Our method involved optimized 2D cultures to induce homogenous otic progenitor cells (OPCs) followed by sequential 3D cultures to develop otic organoids (Fig. 1). SGN-like cells were located on the otic organoid surface and had protein profiles and electrophysiological characteristics similar to those of SGNs in vivo. Neuron-specific labeling (achieved by gene transduction with a viral vector) enabled changes in the morphology of SGN-like cells (including cell body size and neurite structure) to be analyzed in time-lapse images, and the use of this technique provided new insights into the toxic effects of ouabain (which causes SGN-specific damage in animal experiments) and cisplatin (which is ototoxic). Furthermore, a cyclin-dependent kinase-2 (CDK2) inhibitor suppressed the toxic actions of cisplatin on SGN-like cells in otic organoids. The otic organoid described in this study may be a useful model system for drug screening, toxicity testing, and modeling of otic disease in vitro.

## Materials and Methods

### Human iPSC Cultures

hiPSCs were cultured on mitomycin-C-treated murine SNL fibroblast feeder cells on culture dishes coated with 0.1% gelatin. hiPSCs were maintained in standard DMEM/F12 (Sigma-Aldrich) containing 20% KnockOut Serum Replacement (Thermo Fisher Scientific), nonessential amino acids (Sigma-Aldrich), 0.1 mM 2-mercaptoethanol (Sigma-Aldrich), and 4 ng/mL fibroblast growth factor-2 (FGF2; PeproTech) at 37°C with 5% CO_2_. For feeder-free culture conditions, hiPSCs were cultured in StemFit AK02N (Ajinomoto) on iMatrix-511-coated (Nippi) culture dishes. Three hiPSC lines from a healthy 36-year-old female (201B7),^11^ healthy 16-year-old female (WD39),^12^ and healthy ~30-year-old male (648A1)^13^ were used in this study.

### Generation of Otic Organoids

OPC induction from hiPSCs was initiated by exchanging the medium from StemFit (Ajinomoto, Japan) to serum-free (SF) medium. FGF2, FGF3, FGF10, FGF19, and bone morphogenetic protein-4 (BMP4) were added to the SF medium on days 3-5. FGF2, FGF3, FGF10, FGF19, and 8 µM CHIR99021 were added to the SF medium on days 6-8. Differentiated cells were passaged on poly-l-ornithine/fibronectin-coated culture dishes on day 9. OPC medium was exchanged for DMEM/F12 containing 1% N2, 2% B27, 1× Glutamax and 100 mg/mL ampicillin (DFNB medium) and the dishes were kept under hypoxic (5% O_2_) conditions. OPCs were obtained the following day and transferred to 3D conditions to form otic spheroids. Subsequently, neurotrophic factors were added to induce the generation and maturation of otic organoids. Further information is provided in the Supplementary Materials.

### RNA Isolation and Gene Expression Analysis

Total RNA was extracted from OPCs on day 10. PCR amplification of the synthesized cDNA was performed using the primer sets described in Supplementary Table S1. Gene expression assays (qRT-PCR) were carried out using predesigned probes for the OPC markers, paired box-2 (*PAX2*) and *PAX8*. Details of the experimental procedures are provided in the Supplementary Materials.

### Immunofluorescence Analyses

Immunohistochemistry (OPCs and 10-µm sections of otic organoids) and whole-mount immunostaining (otic organoids) were performed using the primary and secondary antibodies listed in Supplementary Table S2. Detailed descriptions of the methods used for immunohistochemistry, whole-mount immunostaining, quantification of cell numbers, measurement of fluorescence intensity, and line-scan analysis of microtubule-associated protein-2 (MAP2) expression are given in the Supplementary Materials.

### Staining of Dead Cells

Dead cells were identified with the terminal deoxynucleotidyl transferase dUTP nick end labeling (TUNEL) assay or by staining with propidium iodide (PI) and Hoechst 33342 (see Supplementary Materials).

### Measurement of Reactive Oxygen Species (ROS) Production

MitoSOX and fluorescence imaging were used to evaluate ROS production in otic organoids (see Supplementary Materials).

### Electrophysiological Recordings

Otic organoids were cultured under four different conditions: 2D, 3D, +NCM, and +BN. Membrane currents and potentials were recorded from bipolar-like cells (2D conditions) or cells transduced with the gene for enhanced green fluorescent protein (EGFP) under the control of the human synapsin-1 promoter (3D, +NCM, and +BN conditions). Adeno-associated virus-5 (AAV5) was used for transduction of the gene for EGFP (AAV-syn-EGFP labeling). Further information is provided in the Supplementary Materials.

### Calcium Imaging

Otic organoids were treated with AAV-syn-GCaMP6s to achieve synapsin-driven expression of the Ca^2+^ indicator, GCaMP6s. Two weeks later, the otic organoids were imaged with a Nipkow disk confocal microscope (excitation wavelength: 488 nm). Further details are provided in the Supplementary Materials.

### Live-cell Imaging

Otic organoids were labeled with AAV-syn-EGFP, and whole-mount live-cell imaging (including optical sectioning and time-lapse imaging) was carried out with a confocal microscope (see Supplementary Materials).

### Drug Administration

Otic organoids labeled with AAV-syn-EGFP and cultured for >45 days were treated with ouabain or cisplatin. The drug was added to the medium, which was changed 48 hours later. The same volume of phosphate-buffered saline (PBS) was administered in control groups.

### Quantification and Statistical Analyses

Statistical analyses were performed using PRISM (GraphPad, USA). Data are expressed as the mean ± SEM. Inter-group comparisons were made using the Student *t* test (two groups) or one-way analysis of variance with the Tukey-Kramer post hoc test (three or more groups).

## Results

### The Optimized Culture Protocol Yielded Cells with High Rates of Expression of OPC Markers

**Figure 2.**
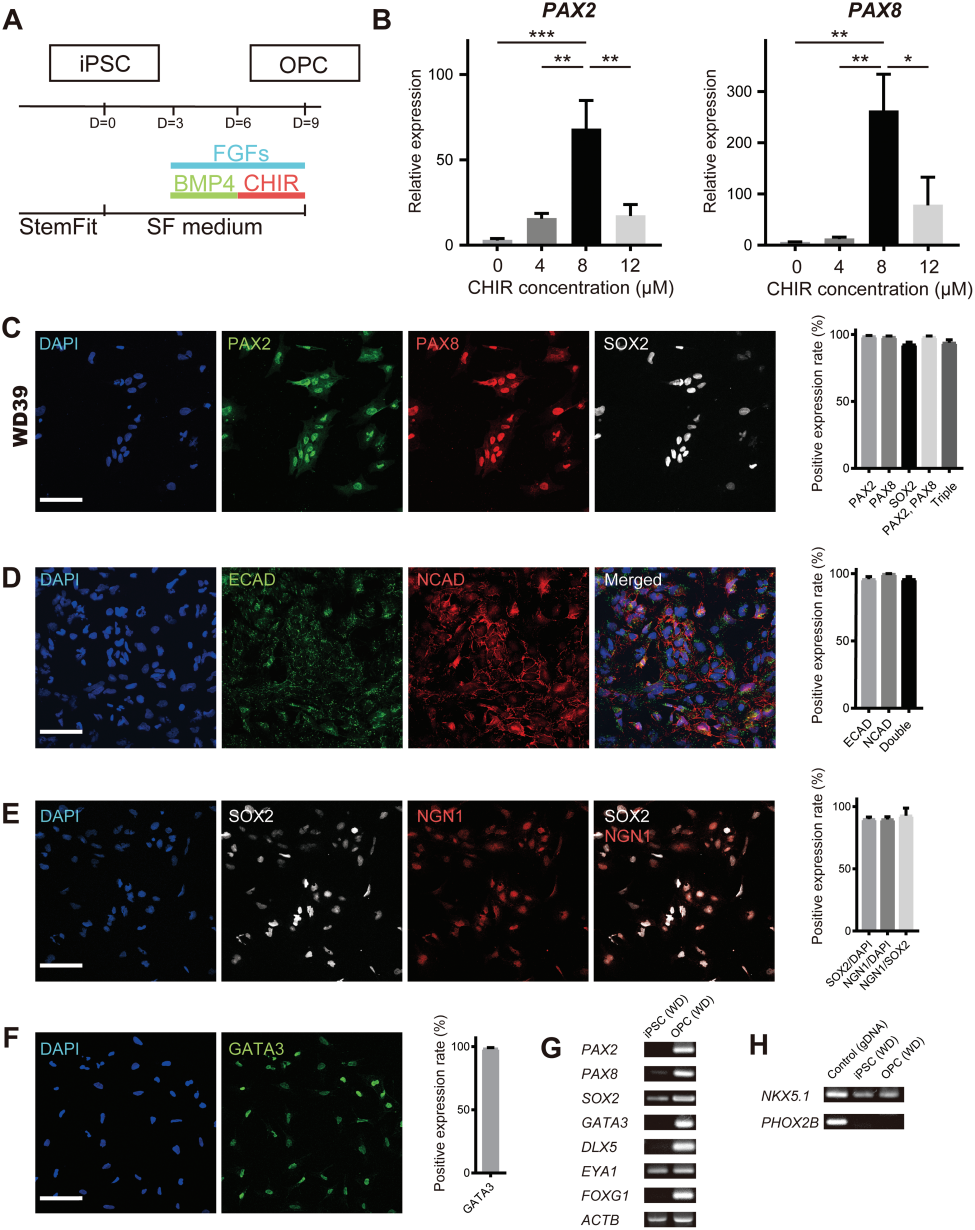
Induction and characterization of OPCs. (A) Schematic diagram of the protocol for OPC induction. At induction, the medium was changed from StemFit to serum-free (SF) medium. FGFs: FGF2, FGF3, FGF10, and FGF19. (B) qRT-PCR analysis of *PAX2* and *PAX8* expression (normalized to *ACTB* expression) in OPCs (WD39) on day 10. Different concentrations of CHIR were applied on days 6-8. Bars show mean ± SEM (*n* = 6 or 7). ∗*P* < .05, ∗∗*P* < .01, ∗∗∗*P* < .001. (C-F) Immunocytochemistry of OPCs derived from hiPSCs (WD39). Cells were stained for OPC markers: PAX2, PAX8, SOX2, E-cadherin (ECAD), N-cadherin (NCAD), NGN1, and GATA3. Scale bars = 100 µm. Quantifications of the positive expression rates are shown to the right of each set of images (mean ± SEM, *n* = 8 for C, *n* = 3 for D-F). (G) RT-PCR analysis of *PAX2*, *PAX8*, *SOX2*, *GATA3*, *DLX5*, *EYA1*, and *FOXG1* expressions in hiPSCs and OPCs. (H) RT-PCR analysis of the expressions of *NKX5.1* (otic marker) and *PHOX2B* (epibranchial marker) in hiPSCs and OPCs. OPCs expressed *NKX5.1* but not *PHOX2B*. Abbreviations: FGFs, fibroblast growth factors; hiPSCs, human-induced pluripotent stem cells; OPCs, otic progenitor cells; qRT-PCR, quantitative reverse transcription-polymerase chain reaction.

OPCs harvested from rodent embryos have the capacity to self-organize and differentiate into inner ear components.^14,15^ We hypothesized that bioengineering a population of homogenous OPCs with comparable biological characteristics to native cells would be key to promoting the self-organization and differentiation of OPCs to establish otic organoids. Therefore, we optimized a previously reported method^16^ that generated OPCs from hiPSCs by the transient addition of FGF2, FGF3, FGF10, FGF19, and BMP4 (Fig. 2A). We also found that the addition of CHIR99021 (CHIR, a glycogen synthase kinase-3 inhibitor) enhanced the expressions of two OPC markers, PAX2 and PAX8. CHIR (added on days 6-8) increased *PAX2* and *PAX8* expressions on day 10 in a concentration-dependent manner, with 8 µM CHIR being the optimal concentration tested (Fig. 2B). Therefore, 8 µM CHIR was included on days 6-8 in all subsequent experiments.

Cell marker expression was evaluated in OPCs derived from three hiPSC lines (WD39, 201B7, and 648A1; obtained from different people). The rate of double expression of PAX2 and PAX8 proteins was comparable between OPCs differentiated from WD39 (98.2 ± 0.7%), 201B7 (97.7 ± 0.8%), and 648A1 (97.0 ± 0.8%) cells (Fig. 2C; Supplementary Fig. S1A). However, the positive expression rate for sex-determining region Y-box-2 (SOX2), a proneurosensory marker, differed between cell lines (WD39, 92.5 ± 1.7%; 201B7, 54.6 ± 5.3%; 648A1, 60.1 ± 5.3%). Additionally, 95.9 ± 1.9% of induced OPCs stained positively for both E-cadherin and N-cadherin (Fig. 2D), which are co-expressed in the otocyst.^17^

SGNs differentiate from overlapping domains positive for Sox2 and neurogenin-1 (Ngn1) in mouse otocysts.^18^ The NGN1 protein expression rate in induced OPCs was 89.8 ± 2.0% in all cells and 92.8 ± 2.3% in SOX2-positive cells (Fig. 2E). GATA3, an otic proneurosensory marker,^19^ was expressed in 98.4 ± 0.8% of all cells (Fig. 2F). RT-PCR confirmed the expressions of other OPC markers, including *DLX5*, *EYA1*, and *FOXG1* (Fig. 2G). Furthermore, *PAX2* and *PAX8* expression levels were higher in OPCs than in parental hiPSCs.

Otic and epibranchial placodes have a close anatomical relationship and both differentiate into neural cells. Induced OPCs showed positive expression for *NKX5.1* (an otic marker) and negative expression for *PHOX2B* (an epibranchial marker)^20,21^ (Fig. 2H). We also confirmed that pluripotency markers (NANOG, SSEA4, TRA-1-60, and TRA-1-81) were downregulated in OPCs (Supplementary Fig. S2).

### OPC Assembly Generated Otic Spheroids That Developed into Otic Organoids

**Figure 3.**
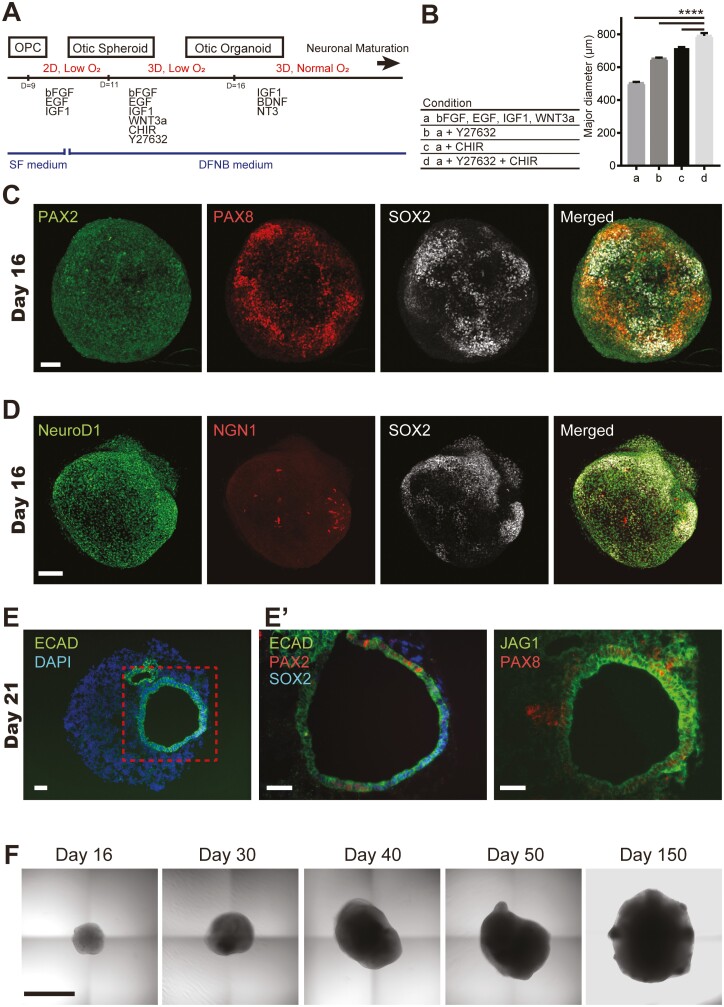
Formation of otic spheroids and development of otic organoids. (A) Schematic diagram of the protocol for otic spheroid formation and otic organoid development. The medium was changed from serum-free (SF) medium to DFNB medium containing DMEM/F12, N2, and B27. (B) The major diameters of otic spheroids cultured under different conditions (days 11-16) were quantified on day 16. Otic spheroid development was improved by co-addition of Y27632 and CHIR (see also Supplementary Fig. S1). Bars show mean ± SEM (*n* = 12). ∗∗∗∗*P* < .0001. (C and D) Whole-mount immunofluorescence images (maximum intensity projections of *z*-stacks) showing PAX2, PAX8, NeuroD1, NGN1, and SOX2 expressions in organoids at day 16. Scale bar = 100 µm. (E) Cryosection of an otic organoid on day 21 showing an otocyst-like structure. The cystic region was positive for E-cadherin (ECAD) and Jagged1 (JAG1). Some cystic epithelium expressed PAX2, PAX8, and SOX2. Magnified views of the boxed area are shown in (E’). Scale bar = 100 µm. (F) Differential interference contrast images illustrating the typical morphology of otic organoids. Scale bar = 1 mm.

Otic spheroids were formed from culturing OPCs under 2D conditions for 2 days (days 9-11) in a hypoxic environment (Fig. 3A). Otic spheroids were then cultured under 3D conditions for 5 days (days 11-16) in a hypoxic environment (Fig. 3A). The otic spheroids became solid tissues under hypoxic conditions while maintaining their progenitor cell characteristics. 3D culture of otic spheroids was performed in the presence of four OPC trophic factors: FGF2, epidermal growth factor (EGF), insulin-like growth factor-1 (IGF1), and Wnt3a. The addition of Y27632 or CHIR during 3D culture suppressed cell death and increased spheroid diameter (Fig. 3B), and the inclusion of both factors produced the largest spheroids with the fewest number of dead cells (Supplementary Fig. S3A, S3B).

Although NGN1 is transiently upregulated in primary neuronal precursors during development, SGN precursors that delaminate from otocysts show downregulated expression of NGN1 and SOX2 as well as upregulated expression of neurogenic differentiation-1 (NeuroD1) which is a downstream mediator of NGN1.^19,22,23^ Consistent with the changes seen in native tissue, otic spheroids at day 16 showed restricted expression of PAX2, PAX8, SOX2 (Fig. 3C), and NGN1 (Fig. 3D) but widespread expression of NeuroD1 (Fig. 3D).

Otic organoids were produced from otic spheroids by 3D culture under normoxic conditions from day 16 (Fig. 3A). Cyst-like structures resembling otocysts were observed in some organoids at around day 20 (Fig. 3E). PAX2, PAX8, and SOX2 were expressed locally in the E-cadherin-positive cystic structure along with Jagged1, a prosensory marker.^24^ The otic organoids maintained their morphological integrity while gradually enlarging in diameter (reaching 2 mm at day 150) when grown in 3D culture (Fig. 3F). We confirmed that stable otic organoids were generated from all three hiPSC lines, but subsequent analyses were performed using organoids derived from WD39 hiPSCs (Supplementary Fig. S3C).

### Otic Organoids Contained Hair Cell-like Cells

Myosin-6 (MYO6)-positive hair cell-like cells surrounded by MAP2-positive neurons were observed within the organoid after 50 days of culture (Supplementary Fig. S4A). POU domain, class 4, transcription factor-3 (POU4F3) was expressed in the nuclei of hair cell-like cells (Supplementary Fig. S4B), and ATOH1 (a hair cell marker) was expressed in POU4F3-positive cells (Supplementary Fig. S4C). Some organoids formed an epithelial structure that included hair cell-like cells (Supplementary Fig. S4D). All 11 organoids immunostained for hair cell markers contained MYO6-positive cells.

### Sensory Neurons in Otic Organoids had Similar Protein Expression Profiles and Morphology to Primary SGNs

**Figure 4.**
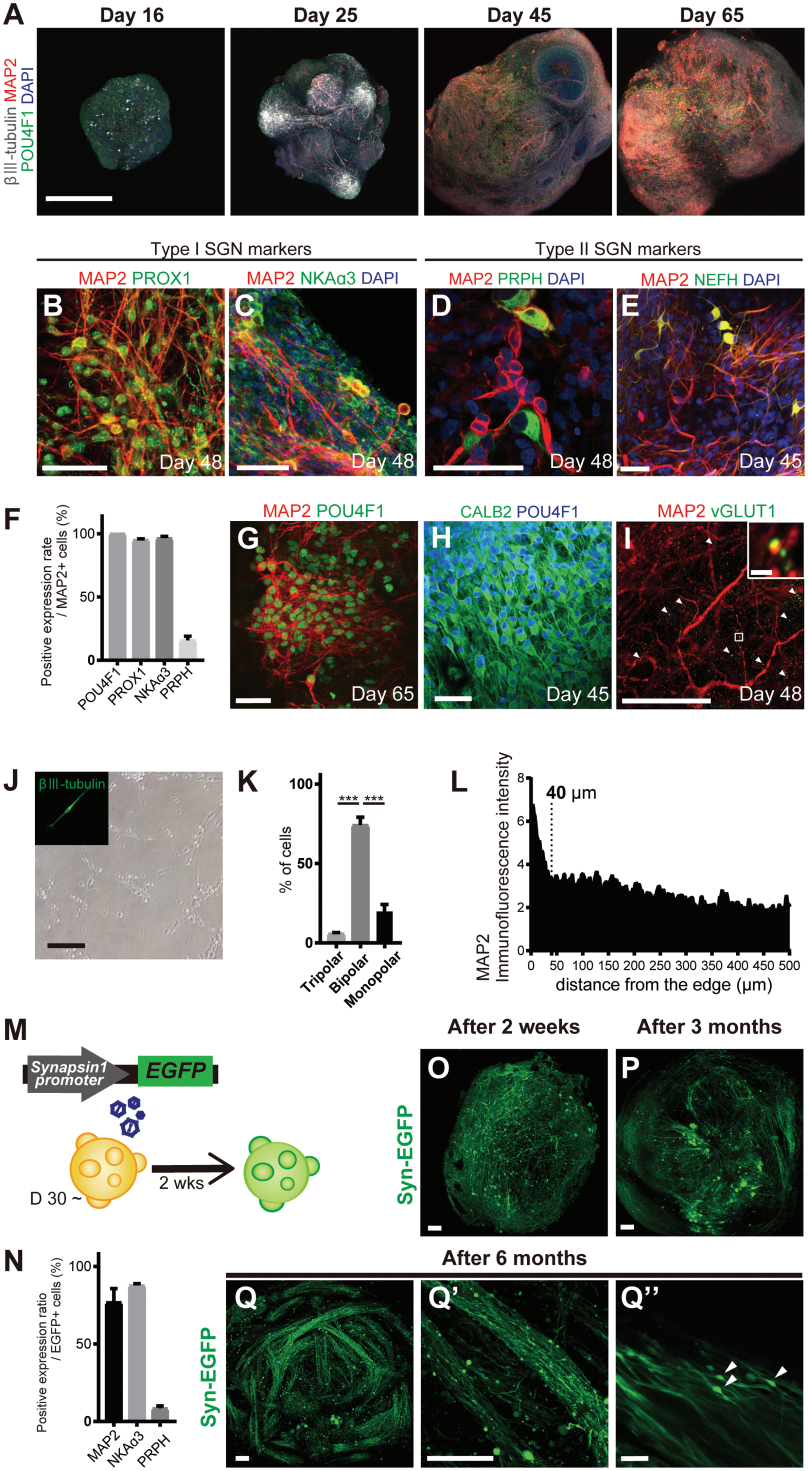
Neuron-like cells on the otic organoid surface resemble SGNs. (A) Whole-mount immunofluorescence images (maximum intensity projections of *z*-stacks) of otic organoids from days 16 to 65. Monitoring of βIII-tubulin and MAP2 expressions revealed the sequential development of sensory neurons. Scale bar = 500 µm. (B-E) Most mature neurons expressed PROX1 and NKAα3 (type I SGN markers). Some mature neurons expressed PRPH and NEFH (type II SGN markers). Scale bar = 50 µm. (F) Quantification of the positive expression rates for POU4F1, PROX1, NKAα3, and PRPH in MAP2-positive cells. Bars show mean ± SEM (*n* = 3). (G and H) Whole-mount immunofluorescence images of otic organoids. Most MAP2-positive mature neurons expressed POU4F1 and CALB2. Scale bar = 50 µm. (I) vGLUT1 localized to MAP2-positive dendrite-like structures. Scale bar = 50 µm. (J) Bright-field image of neurons from a dissociated otic organoid. Upper-left window: immunostaining for βIII-tubulin. Scale bar = 100 µm. (K) The number of dendrites for each neuron. Many neurons were bipolar. Scale bar, 100 µm. Data are presented as mean ± SEM (*n* = 3). ∗∗∗*P* < 0.001. (L) Line-scan analyses of organoid cryosections immunostained for MAP2. For each of the three organoids, four lines were analyzed from the edge to the center. Fluorescence intensity was normalized to that of a background region. (M) Schematic diagram showing the labeling of otic organoids with EGFP for live-cell imaging. (N) Quantification of the positive expression rates for MAP2, NKAα3, and PRPH in GFP-positive neurons. Note that 76.8% of GFP-positive cells were stained for MAP2, indicating that they were mature neurons, and both type I and type II SGN-like cells were labeled. Bars show mean ± SEM (*n* = 3). (O-Q) Live-cell imaging of labeled organoids (maximum intensity projections of *z*-stacks). GFP-positive neurons were initially scattered throughout the organoid but were aligned in bundles after 6 months (Q’). The bundles contained cell bodies (arrowheads) and neurites (Q’’). Scale bars = 100 µm (O-Q, Q’) and 50 µm (Q’’). Abbreviations: EGFP, enhanced green fluorescent protein; GFP, green fluorescent protein; MAP2, microtubule-associated protein-2; NEFH, neurofilament heavy chain; PRPH, peripherin; SGNs, spiral ganglion neurons.

POU4F1 is a peripheral sensory neuron marker expressed in the primordial vestibulocochlear ganglion and continues to be expressed after differentiation of the spiral and vestibular ganglia.^25^ βIII-tubulin and MAP2 are markers of immature and mature neurons, respectively.^26,27^ Therefore, we analyzed the expressions of these markers in organoids from days 16 to 65 to confirm neuronal maturation (Fig. 4A). POU4F1 protein was detected across a broad part of the organoid surface on day 16, and its expression was maintained up to day 65. Only a few cells stained positively for βIII-tubulin on day 16, but βIII-tubulin-positive cells with a fibrous morphology were identified in protruding regions of the organoid on day 25. MAP2 expression was first observed on day 25, and the entire organoid surface was covered with MAP2-positive cells on days 45 and 65. These results indicate that the organoid surface contained neurons that matured after around 45 days of culture. Thus, all subsequent analyses used organoids cultured for >45 days, and the mature neurons in these organoids were considered to be induced SGNs (iSGNs).

The characteristics of MAP2-positive iSGNs on the organoid surface were analyzed using whole-mount immunohistochemistry. Two types of SGNs are recognized (type 1 and type 2) according to their function and connectivity to hair cells, and recent reports have revealed the presence of three subtypes of type 1 SGNs.^28,29^ Most iSGNs were positive for prospero-related homeobox-1 (PROX1) and Na/K-ATPase isoform α3 (NKAα3), which are markers of type 1 SGNs,^19^ and some were positive for peripherin (PRPH) and neurofilament heavy chain (NEFH), which are markers of type 2 SGNs^30,31^ (Fig. 4B–4E). These findings indicate that the organoids contained two types of SGN-like neurons, with type 1 predominating (Fig. 4F). We also analyzed whether subtype specification of type 1 SGNs had occurred. Most MAP2-positive cells expressed POU4F1 (Fig. 4F, 4G) and calbindin-2 (CALB2; Fig. 4H), indicating that the subtype identities of type 1 SGN-like neurons had not emerged during culture.^28^ Interestingly, puncta expressing vesicular glutamate transporter-1 (vGLUT1) were detected in MAP2-positive dendrite-like structures, suggesting that the iSGNs had formed glutamatergic synapses (Fig. 4I).

Cells dissociated from organoids on day 20 were cultured under 2D conditions to observe the morphology of the neuronal processes. Most neurons were bipolar (Fig. 4J, 4K), as are native SGNs.

### Live-cell Imaging of Organoids Containing a Neuron-specific Label

Otic organoids are well suited to live-cell imaging of their iSGNs because the mature neurons are mainly located within 40 µm of the edge of the organoid (Fig. 4L). Therefore, we evaluated 30-day organoid cultures 2 weeks after the use of AAV5 to transduce the organoid cells with the EGFP gene under the control of the human synapsin-1 promoter (AAV-syn-EGFP labeling) (Fig. 4M). Immunohistochemistry revealed that most EGFP-positive cells expressed MAP2 (76.8 ± 8.9%) and that NKAα3-positive and PRPH-positive iSGNs comprised 87.7 ± 1.2% and 8.4 ± 1.6% of the EGFP-positive cells, respectively (Fig. 4N).

The labeled iSGNs exhibited time-dependent fascicle formation. Labeled neurons were sporadically present on the organoids at 2 weeks after AAV-syn-EGFP labeling (Fig. 4O) but became aligned by 3 months (Fig. 4P) and formed fascicles by 6 months (Fig. 4Q). The fascicles contained neuronal cell bodies and neurites (Fig. 4Qʹ, Q″).

### The Neuron-like Electrophysiological Properties of a Subset of Otic Organoid Cells Varied Depending on the Culture Conditions

**Figure 5.**
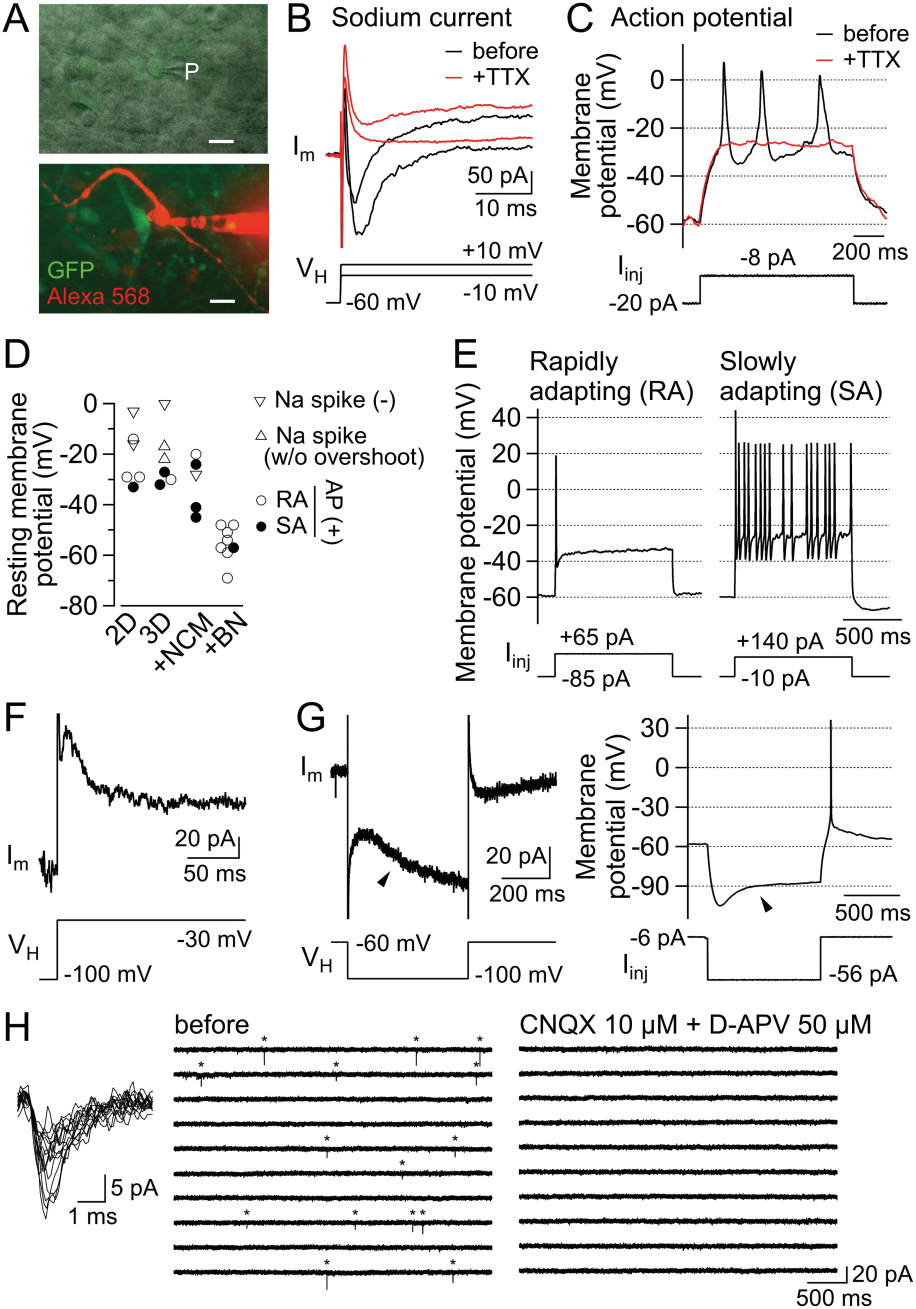
Neuron-like electrophysiological properties of otic organoid cells. (A) Micrographs of an organoid surface. Upper panel: bright-field image with oblique illumination (monochrome) showing GFP fluorescence. P, tip of the recording pipette. Lower panel: image showing GFP and Alexa Fluor 568 fluorescence. Scale bar = 10 µm. (B) Representative traces from a GFP-positive cell showing membrane current (I_m_) recorded in response to depolarizing steps (V_H_) in the absence (black) and presence (red) of TTX. Note the presence of TTX-sensitive inward currents. See also Supplementary Fig. S5A. (C) Representative traces showing the membrane potential changes in response to depolarizing current injections (I_inj_) in the absence (black) and presence (red) of TTX. Note the generation of TTX-sensitive action potential-like spikes. See also Supplementary Fig. S5B. (D) Resting membrane potential of cells cultured under distinct conditions. Markers indicate the electrophysiological response of each cell: Na spike (-), cells without an action potential (AP) or inward current in response to depolarization; Na spike (w/o overshoot), cells with action potential-like spikes that did not reach 0 mV; AP (+), cells with overshooting action potentials classified as either rapidly adapting (RA) or slowly adapting (SA) (see Fig. 5E). (E) Action potentials elicited by the injection of depolarizing current in two representative GFP-positive cells with rapidly adapting (left) and slowly adapting (right) firing properties. See also Supplementary Fig. S5E. (F) Example of a transient outward current (I_m_) elicited in response to depolarization (V_H_) in a GFP-positive cell. See also Supplementary Fig. S5F. (G) Representative recordings from the same GFP-positive cell showing the membrane current response to a hyperpolarizing step (left) and the membrane potential response to an injection of hyperpolarizing current (right). Note the slowly activating, non-inactivating inward current (arrowhead, left panel) and a sag-rebound in membrane potential (arrowhead, right panel). See also Supplementary Fig. S5G. (H) Spontaneous postsynaptic currents recorded from a GFP-positive cell in an organoid. Left panel: overlaid traces showing the spontaneously occurring postsynaptic currents (*n* = 15 events) indicated with asterisks in the middle panel. Right panel: spontaneous events were abolished in the co-presence of CNQX and D-APV. Traces in the middle and right panels represent a continuous 40-second recording from top-left to bottom-right. Abbreviations: CNQX, 6-cyano-7-nitroquinoxaline-2,3-dione; D-APV, d-2-amino-5-phosphonovaleric acid; GFP, green fluorescent protein; TTX, tetrodotoxin.

To identify appropriate culture conditions for neuronal maturation, we investigated the electrophysiological properties of neuron-like cells in organoids prepared under different conditions: (1) 2D culture of dispersed organoids in DFNB medium containing IGF1 (2D); (2) 3D culture of organoids in DFNB medium containing IGF1 (3D); (3) culture of organoids in Neuron Culture Medium (NCM, Wako) for at least 1 week before electrophysiology experiments (+NCM); and (4) culture of organoids in medium containing brain-derived neurotrophic factor (BDNF) and neurotrophin-3 (NT3) from day 16 of culture (+BN). Membrane currents were recorded from neuron-like EGFP-positive cells in the otic organoids (Fig. 5A). Whole-cell recordings enabled the injection of Alexa Fluor 568 into the cell to observe its morphology (Supplementary Fig. S5A). Cells from which recordings were made were characterized by long, thin, dendrite-like processes arising from the soma; longer neurites and branching neurites were observed only in cells from organoids cultured under +BN conditions (Supplementary Fig. S5A).

Depolarizing voltage steps from −60 mV elicited a transient tetrodotoxin (TTX)-sensitive inward current in most neuron-like cells (*n* = 19/20; Fig. 5B; Supplementary Fig. S5B), suggesting that the majority of these cells expressed voltage-gated fast sodium channels. A rapid depolarization from −60 mV in current-clamp recordings elicited overshooting TTX-sensitive action potentials in most cells (*n* = 21/25; Fig. 5C; Supplementary Fig. S5C), consistent with a neuronal phenotype. Furthermore, TTX-sensitive action potentials were observed under all four culture conditions (*n* = 4/6 for 2D culture, 3/6 for 3D culture, 4/5 for +NCM culture, and 8/8 for +BN culture; Fig. 5D).

Mean resting membrane potential (Fig. 5D) was comparable between neurons cultured under 2D (−21 ± 4.7 mV, *n* = 6), 3D (−22 ± 4.1 mV, *n* = 6) or +NCM (−35 ± 4.9 mV, *n* = 5) conditions but significantly more negative for neurons cultured under +BN conditions (−55 ± 2.4 mV, *n* = 8; 2D vs +BN, *P* < .001; 3D vs +BN, *P* < .001; +NCM vs +BN, *P* = .005; ANOVA and Tukey-Kramer test). Neurons cultured under +BN conditions had action potentials with a higher amplitude, shorter half-width, greater slope, and larger after-hyperpolarization than neurons cultured under the other conditions likely due to the more negative resting membrane potential in neurons cultured under +BN conditions (Supplementary Fig. S5D). Irrespective of the culture condition, continuous depolarization elicited two distinct patterns of firing (Fig. 5D, 5E; Supplementary Fig. S5E) similar to those reported in rapidly adapting and slowly adapting SGNs isolated from the mouse.^32^

Depolarization-activated transient outward current, which has been described in rat and guinea pig isolated SGNs,^33,34^ was observed in 1 of 8 neurons cultured under +BN conditions (Fig. 5F; Supplementary Fig. S5F). Hyperpolarization-activated inward current and hyperpolarizing sag and rebound, which have been described in mouse and guinea pig isolated SGNs,^35,36^ were observed in all 8 neurons cultured under +BN conditions (Fig. 5G; Supplementary Fig. S5G). Thus, SGN-like cells cultured under +BN conditions expressed voltage-dependent ion channels commonly found in mature SGNs, implying that BDNF and NT3 promoted the maturation of neuron-like cells during organoid culture.

Neuronal excitability leads to action potential-dependent activation of voltage-gated Ca^2+^ channels, which is a key process underlying transmitter release. Therefore, we visualized intracellular Ca^2+^ transients using synapsin-driven expression of the Ca^2+^ indicator, GCaMP6s. The application of extracellular solution containing 20 mM or 40 mM K^+^ elicited a robust rise in the Ca^2+^-dependent fluorescence of both the somata and processes (Supplementary Fig. S6; Supplementary Videos S1 and S2). As depolarization caused by 20 mM K^+^ is insufficient to activate high-threshold voltage-dependent Ca^2+^ channels,^37^ it is likely that the cells responding to this high-K^+^ solution had neuronal excitability.

Notably, iSGNs showed signs of functional synapse formation. Recordings from most neurons demonstrated the frequent occurrence of spontaneous, small-amplitude (~10-15 pA) inward current events (marked ∗ in Fig. 5H) with a rapidly rising phase and an exponentially decaying phase. These events are characteristic of excitatory postsynaptic currents (EPSCs) reported in mature neurons. It is likely that these EPSCs were mediated by glutamate release and activation of ionotropic glutamate receptors, as they were abolished by 6-cyano-7-nitroquinoxaline-2,3-dione (CNQX), an AMPA/kainate receptor antagonist, and d-2-amino-5-phosphonovaleric acid (D-APV), a non-*N*-methyl-d-aspartate (NMDA) receptor antagonist (Fig. 5H).

### Na/K-ATPase Inhibition Induced Cell Body Expansion and Death of iSGNs

**Figure 6.**
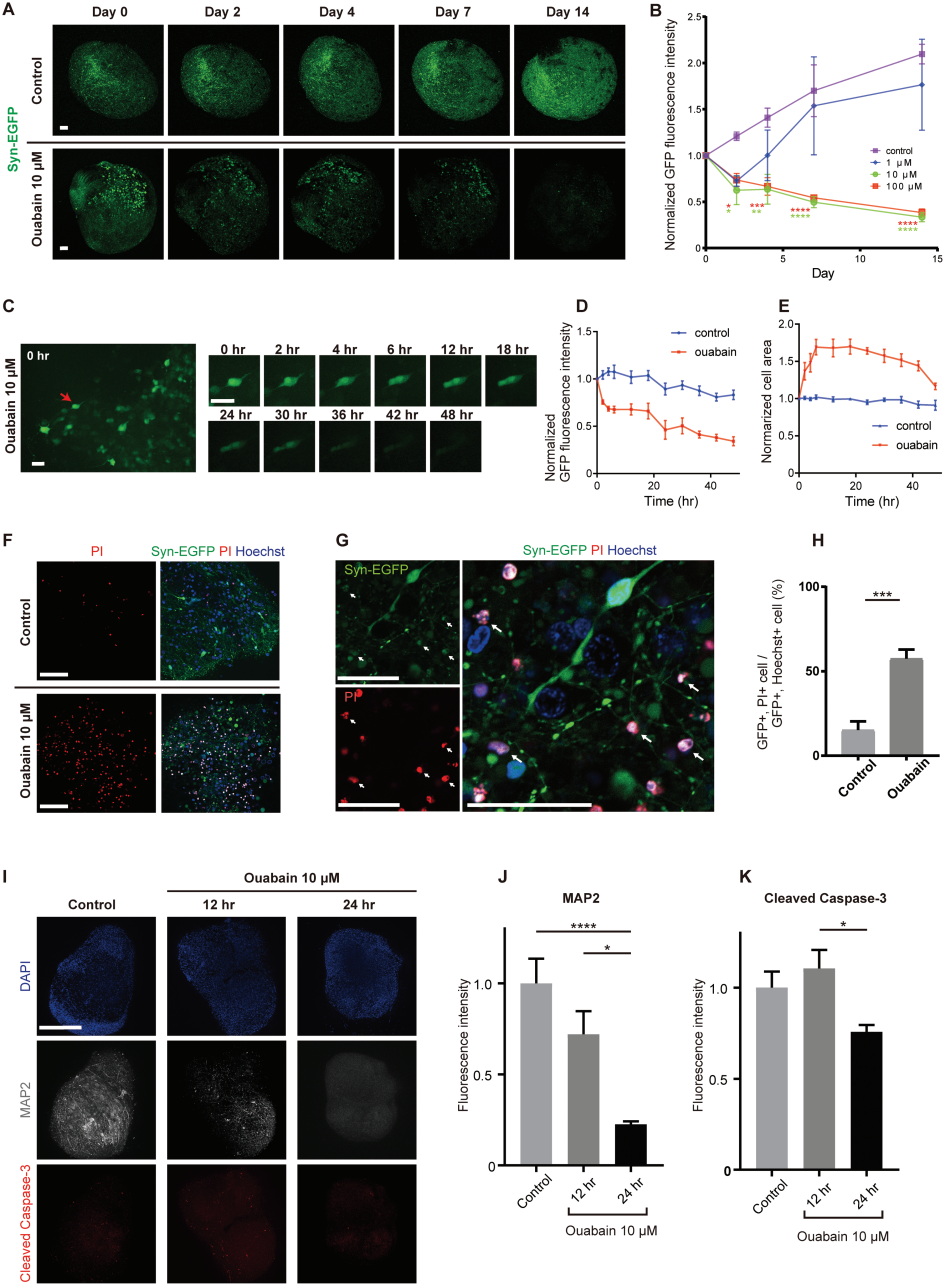
Na/K-ATPase inhibition induced cell expansion followed by the death of iSGNs. (A) Sequential live-cell images (maximum intensity projections of *z*-stacks) obtained from labeled organoids (with/without 10 µM ouabain) over a 14-day period. Scale bar = 100 µm. See also Supplementary Fig. S7. (B) Temporal changes in GFP fluorescence intensity (normalized to the value at day 0) in organoids treated with different concentrations of ouabain. Note that 10 µM ouabain was sufficient to induce cell damage and decrease GFP fluorescence intensity. Data shown as mean ± SEM (*n* = 3). ∗*P* < .05, ∗∗*P* < .01, ∗∗∗*P* < .001, ∗∗∗∗*P* < .0001. (C) Time-lapse images of labeled organoids. At each time point, the optically sectioned image (*z*-stack) was converted to a full-focus image. The images in the right panel were obtained from the cell indicated by an arrow in the left panel. Scale bar = 50 µm. See also Supplementary Videos S3 and S4. (D and E) GFP fluorescence intensity and cell area for 3 representative cells selected from organoids exposed or not exposed to 10 µM ouabain. Data presented as mean ± SEM (*n* = 3). See also Supplementary Fig. S8. (F) Confocal images of labeled organoids (48 hours after exposure to 10 µM ouabain) stained with propidium iodide (PI) and Hoechst. The nuclei of dead cells were stained by PI. Scale bar, 100 μm. (G) Representative images of organoids exposed to ouabain. Cells positive for both GFP and PI indicate dead neurons. Scale bar = 50 µm. (H) Proportions of GFP/PI double-positive cells and GFP/Hoechst double-positive cells in organoids administered or not administered ouabain. Bars show mean ± SEM (*n* = 3). ∗∗∗*P* < .001. (I) Representative confocal images of organoids double-stained for MAP2 and cleaved caspase-3. Ouabain-treated organoids were harvested at 12 hours or 24 hours after drug administration. Scale bar = 500 µm. (J) MAP2 immunofluorescence intensity analyzed in untreated organoids (Control) or organoids treated with ouabain for 12 hours (12 hr) or 24 hours (24 hr). Bars show mean ± SEM (*n* = 6). ∗*P* < 0.05, ∗∗∗∗*P* < 0.0001. (K) Cleaved caspase-3 immunofluorescence intensity analyzed in untreated organoids (Control) or organoids treated with ouabain for 12 hours (12 hr) or 24 hours (24 hr). Bars show mean ± SEM (*n* = 6). ∗*P* < 0.05. Abbreviations: GFP, green fluorescent protein; iSGNs, induced spiral ganglion neurons; MAP2, microtubule-associated protein-2.

Ouabain, a Na/K-ATPase inhibitor, is widely used in animal models to ablate SGN cells without damaging hair cells,^3^ and the neuropathic effects of ouabain have been analyzed in ex vivo assays.^38,39^ We investigated whether iSGNs reacted to ouabain like native SGNs. In control organoids labeled with AAV-syn-EGFP, fluorescence increased during the subsequent 2-week period due to EGFP accumulation. However, fluorescence decreased over the 2-week period in organoids exposed to 10 µM or 100 µM ouabain (Sigma-Aldrich) (Fig. 6A, 6B; Supplementary Fig. S7). The effects of ouabain on single iSGNs were evaluated from magnified images of EGFP-positive bipolar neurons obtained hourly (Fig. 6C; Supplementary Fig. S8, Supplementary Videos S3 and S4). Compared to control iSGNs, ouabain-treated iSGNs exhibited a progressive decrease in fluorescence intensity from 2 hours after drug administration and an increase in cell area that peaked at 6 hours before gradually decreasing (Fig. 6D, 6E).

Staining with PI and Hoechst 33342 was used to elucidate whether the morphological changes observed 48 hours after ouabain administration were attributable to cell death or transient cell damage (Fig. 6F, 6G). More PI-positive cells were observed in ouabain-treated organoids than in controls, suggesting that ouabain caused iSGN death (Fig. 6H). Although ouabain-induced apoptosis of native SGNs has been reported,^38,39^ the cell body expansion observed in our experiments was not consistent with conventional apoptotic processes such as pyknosis. Therefore, we performed whole-mount immunohistochemistry to analyze the expressions of MAP2 and cleaved caspase-3 in cells on the organoid surface (Fig. 6I). Exposure to ouabain led to a decrease in MAP2 expression within 24 hours (Fig. 6J) but little or no upregulation of cleaved caspase-3 (Fig. 6K), implying that pathways other than apoptosis may have contributed to ouabain-induced iSGN death.

### A CDK2 Inhibitor Suppressed Cisplatin-induced Apoptosis of iSGNs

**Figure 7.**
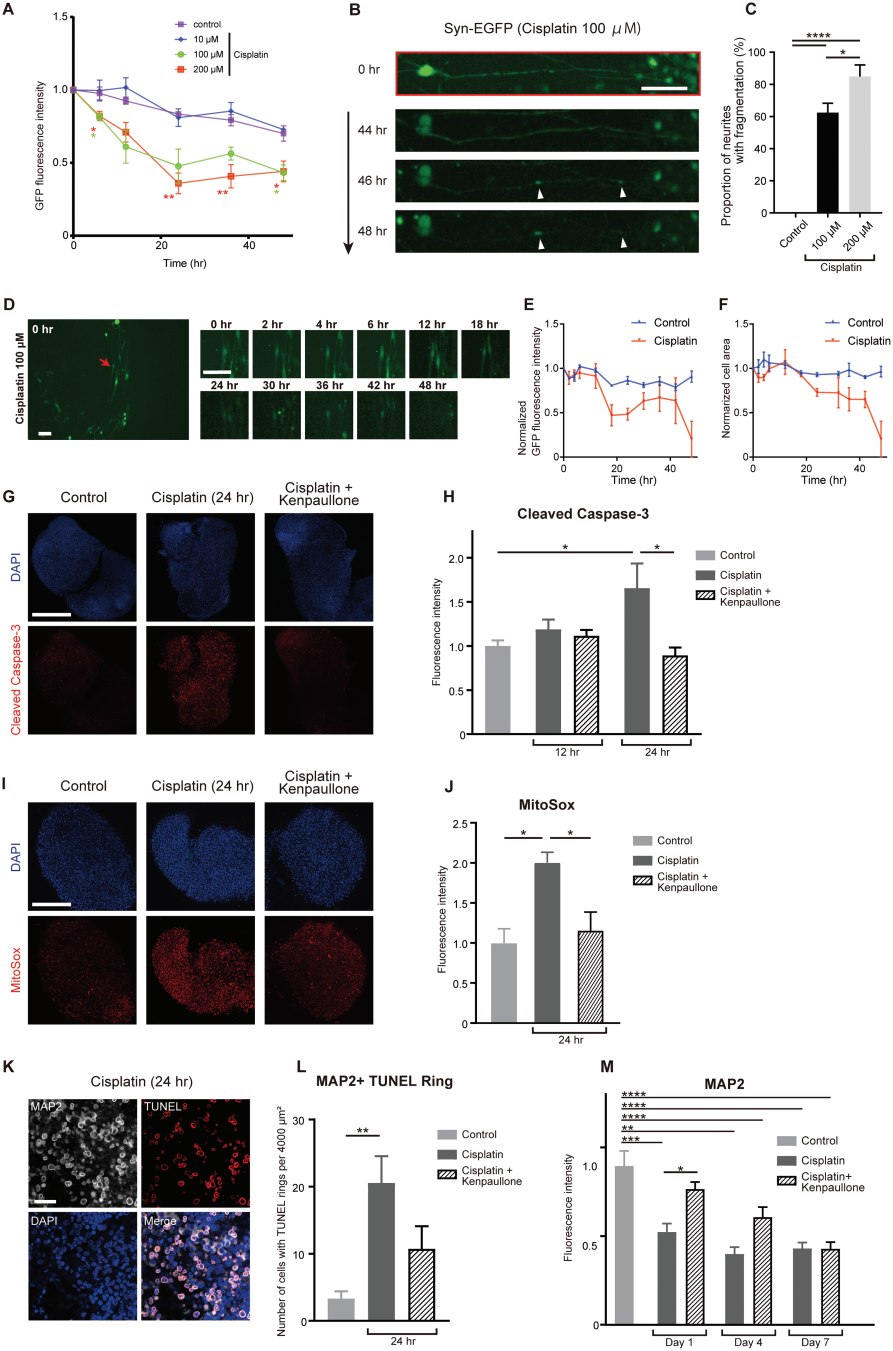
A CDK2 inhibitor protected against cisplatin-induced iSGN apoptosis. (A) GFP fluorescence intensity in the entire field of view was analyzed from time-lapse images of labeled organoids over a 48-hour period. Various concentrations of cisplatin (10-200 µM) were administered. Data presented as mean ± SEM (*n* = 5 or 6). ∗*P* < .05, ∗∗*P* < .01. (B) Representative images showing neurite fragmentation after the administration of 100 µM cisplatin. Arrowheads indicate fragmentation points on the neurite. Scale bar = 50 µm. (C) Proportion of neurites in the field of view showing fragmentation during a 48-hour period. No neurites exhibited fragmentation in the control and 10 µM cisplatin groups. Bars show mean ± SEM (*n* = 5 or 6). ∗*P* < .05, ∗∗∗∗*P* < .0001. (D) Time-lapse images of labeled organoids. The optically sectioned image (*z*-stack) was converted to a full-focus image. Images in the right panel were obtained from the cell indicated by an arrow in the left panel. Scale bar = 50 µm. See also Supplementary Videos S5 and S6. (E and F) GFP fluorescence intensity and cell area for 3 representative cells selected from organoids administered or not administered 100 µM cisplatin. Data presented as mean ± SEM (*n* = 3). See also Supplementary Fig. S9. (G) Representative confocal images of organoids immunostained for cleaved caspase-3. Organoids were harvested 24 hours after treatment with cisplatin (100 µM) with or without kenpaullone (a CDK2 inhibitor). Scale bar = 500 µm. (H) Cleaved caspase-3 immunofluorescence intensity in untreated organoids (Control) and organoids treated with cisplatin with/without kenpaullone for 12 hours (12 hr) or 24 hours (24 hr). Bars show mean ± SEM (*n* = 6). ∗*P* < .05. (I) Representative confocal images of organoids stained with MitoSOX. Organoids were harvested 24 hours after treatment with cisplatin (100 µM) with/without kenpaullone. Scale bar = 500 µm. (J) MitoSOX fluorescence intensity in untreated organoids (Control) and organoids treated with cisplatin with/without kenpaullone for 24 hours (24 hr). Bars show mean ± SEM (*n* = 6). ∗*P* < .05. (K) Representative confocal images of TUNEL rings in a cisplatin-treated organoid. TUNEL rings in MAP2-positive cells indicate apoptotic neurons. Scale bar = 50 µm. (L) Number of TUNEL rings in MAP2-positive cells per 4000 µm^2^ of organoid (harvested 24 hours after drug administration). Bars show mean ± SEM (*n* = 6). ∗∗*P* < .01. (M) Organoids treated with cisplatin with/without kenpaullone were harvested at day 1, 4, or 7 and immunostained for MAP2. Bars show mean ± SEM (*n* = 6). ∗*P* < .05, ∗∗*P* < .01, ∗∗∗*P* < .001, ∗∗∗∗*P* < .0001. Abbreviations: CDK2, cyclin-dependent kinase-2; GFP, green fluorescent protein; iSGN, induced spiral ganglion neurons; MAP2, microtubule-associated protein-2; TUNEL, terminal deoxynucleotidyl transferase dUTP nick end labeling.

Cisplatin is a widely used chemotherapeutic drug that can induce hearing loss as an adverse effect due to damage to the organ of Corti, stria vascularis, and SGNs.^40^ 100 µM cisplatin (Yakult, Japan) decreased the fluorescence intensity of AAV-syn-EGFP-labeled organoids (Fig. 7A), indicating a cytotoxic effect. Furthermore, 100 µM cisplatin caused neurite fragmentation (Fig. 7B, 7C) and decreases in the fluorescence intensity and cell area of individual cells (Fig. 7D–7F; Supplementary Fig. S9) that would be consistent with apoptotic cell death.

Whole-mount immunohistochemistry experiments revealed that the organoids exhibited upregulation of cleaved caspase-3 at 24 hours after cisplatin administration (Fig. 7G, 7H). Kenpaullone (Sigma-Aldrich), a CDK2 inhibitor reported to protect against cisplatin-induced hair cell damage,^41^ prevented the upregulation of cleaved caspase-3 by cisplatin (Fig. 7G, 7H; 5 μM of kenpaullone was applied 1 hour before cisplatin). Since mitochondrial production of ROS is associated with cisplatin-induced ototoxicity,^42^ we next examined whether ROS production was involved in the effects of cisplatin and kenpaullone. Cisplatin-treated organoids exhibited an increase in the fluorescence intensity of MitoSOX, an indicator of mitochondrial superoxide, and kenpaullone inhibited this effect of cisplatin (Fig. 7I, 7J).

MAP2 staining and the TUNEL assay were used in combination to evaluate the effects of cisplatin and kenpaullone specifically on iSGNs. The TUNEL assay produces annular staining (a “TUNEL ring”) during the early phase of apoptosis,^43^ hence the presence of TUNEL rings in MAP2-positive cells was used to indicate apoptotic changes in iSGNs (Fig. 7K). Cisplatin-treated organoids contained significantly more MAP2-positive cells with TUNEL rings than controls, and there was a tendency for kenpaullone to inhibit this effect of cisplatin, although statistical significance was not attained (*P* = .084; Fig. 7L). In additional experiments, kenpaullone was found to significantly inhibit the cisplatin-induced decrease in MAP2 fluorescence intensity at day 1 but not at days 4 or 7 (Fig. 7M), suggesting that kenpaullone initially protected against cisplatin-induced neural damage but ultimately did not prevent cell death under these conditions.

## Discussion

This study describes a new protocol for sequential 2D/3D culture that generates differentiated otic organoids with SGN-like cells on their surface with an efficiency exceeding 90% (data not shown). In our view, the key to achieving highly efficient induction of otic organoids was to generate homogenous OPCs in 2D culture before initiating self-organization in 3D culture. 2D culture is preferable to 3D culture for OPC induction because it allows more uniform exposure of cells to recombinant proteins or small compounds that manipulate cell fate. To generate homogenous OPCs, we modified a protocol described by Hosoya et al (2017). The addition of CHIR likely compensated for deficient endogenous Wnt signaling. Indeed, a study of mouse iPSC-derived inner ear organoids revealed that OPCs were induced more efficiently when CHIR was included prior to otic placode formation.^44^

SGN-like cells acquired functional as well as genetic and morphological neuronal phenotypes, including: (1) a stable, strongly negative resting membrane potential; (2) TTX-sensitive action potentials in response to depolarization (observed in 84% of the cells examined); (3) increases in intracellular Ca^2+^ levels in the soma and processes in response to mild depolarization induced by an elevation of extracellular K^+^ concentration; (4) neuron-specific voltage- and time-dependent currents similar to those reported in rodent SGNs^33-36^; and (5) two distinct firing patterns reminiscent of the cochlear position-dependent firing patterns in mouse isolated SGNs.^32^ Furthermore, these cells exhibited postsynaptic currents mediated by synaptically released glutamate, suggesting that a subset of neuron-like cells had formed synapses. Therefore, neurons differentiating into primary sensory cells likely form synapses with SGN-like neurons and send them excitatory information within the organoid. Based on the results of the electrophysiology experiments, we were able to optimize the culture conditions to achieve neuronal maturation.

Although SGN-like cells had similar protein expression profiles, morphological characteristics and electrophysiological properties to native SGNs, we could not conclude whether these cells developed as SGNs or vestibular ganglion neurons (VGNs). Lu et al used gene microarrays to compare expression patterns between murine SGNs and VGNs during development and found higher expressions of Prox1 and Zfpm2 in SGNs than in VGNs at the E12 to P15 stage.^45^ The iSGNs examined in this study were mostly positive for PROX1, suggesting that they had physiological similarities to SGNs. Additionally, the protocol of Hosoya et al (2017) successfully induced cochlear outer sulcus cells, implying that the OPCs had already deviated to a cochlear lineage; this could be taken as indirect evidence of the formation of SGN-like neurons in our study.

Labeling of otic organoids with AAV-syn-EGFP permits the sequential observation of a group of cells or an individual neuron to evaluate the actions of a drug. We examined the effects of two drugs (ouabain and cisplatin) reported to induce apoptosis in SGNs. Although we observed cisplatin-induced cell death that was indicative of apoptosis (as described previously for native SGNs), the mechanism underlying ouabain-induced cell death appeared to differ from that reported for native SGNs.

Ouabain inhibits the Na/K-ATPase that generates Na^+^ and K^+^ gradients between the intracellular and extracellular spaces and contributes to the regulation of cell body volume and intracellular osmotic pressure. Ouabain is widely used to create animal models of SGN-specific ablation.^3^ Ouabain-induced SGN death is reportedly due to an apoptotic-like process caused by the blocking of NKAα3.^38,39^ Fu et al (2013) demonstrated that exposure of rat cultured SGNs to ouabain increased the number of annexin V-positive cells, but gene array analysis revealed that some pro-apoptotic genes were downregulated. In our study, ouabain-treated iSGNs showed cell expansion rather than pyknosis and only a slight upregulation of cleaved caspase-3, which is not consistent with typical apoptosis. Although it is possible that we observed a pre-apoptotic state, other mechanisms of cell death need to be considered. For example, a progressive increase in osmotic pressure due to Na/K-ATPase inhibition may have induced excessive cell expansion that led to cell death. Further research is needed to elucidate the effects of ouabain on human SGNs.

Cisplatin is widely used to treat solid malignant tumors but causes ototoxicity in around 60% of patients.^46^ Cisplatin-induced hearing loss is thought to be due to excessive ROS production in cochlear cells, but a definitive mechanism has yet to be elucidated. We found that cisplatin-induced iSGN apoptosis and mitochondrial ROS production. Moreover, kenpaullone, a CDK2 inhibitor, suppressed the ototoxic effects of cisplatin in the short term but did not prevent cell death in the long term under our experimental conditions. Our findings are broadly consistent with previous research demonstrating that kenpaullone protected against cisplatin-induced cochlear hair cell death in zebrafish and rodents.^41^ Nevertheless, how cisplatin increases the activity of CDK2 and how this leads to enhanced mitochondrial ROS production remain unknown.^41^ Further experiments are required to confirm the protective effect of kenpaullone against cisplatin-induced SGN injury and establish the underlying mechanisms.

In conclusion, we have described a new protocol for generating otic organoids from hiPSCs and evaluated the effects of drugs on iSGNs located on the surface of these organoids. Although mouse dissociated SGNs have been used for drug screening,^47^ our study is the first to describe the use of human iSGNs to evaluate the effects of drugs. Our experimental platform based on hiPSC-derived iSGNs in otic organoids is a candidate novel drug screening system that provides a similar physiological environment to that experienced by SGNs in situ.

## Supplementary Material

szab023_suppl_Supplementary_Figure_S1

szab023_suppl_Supplementary_Figure_S2

szab023_suppl_Supplementary_Figure_S3

szab023_suppl_Supplementary_Figure_S4

szab023_suppl_Supplementary_Figure_S5

szab023_suppl_Supplementary_Figure_S6

szab023_suppl_Supplementary_Figure_S7

szab023_suppl_Supplementary_Figure_S8

szab023_suppl_Supplementary_Figure_S9

szab023_suppl_Supplementary_Tables

szab023_suppl_Supplementary_Materials

szab023_suppl_Supplementary_Video_S1

szab023_suppl_Supplementary_Video_S2

szab023_suppl_Supplementary_Video_S3

szab023_suppl_Supplementary_Video_S4

szab023_suppl_Supplementary_Video_S5

szab023_suppl_Supplementary_Video_S6

## Data Availability

The data that support the findings of this study are available from the corresponding author upon reasonable request.
