# Supplementary figures and images for: Otic Organoids Containing Spiral Ganglion Neuron-like Cells Derived from Human-induced Pluripotent Stem Cells as a Model of Drug-induced Neuropathy
Source: Stem Cells Transl Med. 2022 Mar 7;11(3):282–96. doi: 10.1093/stcltm/szab023 (PMC8968745; doi:10.1093/stcltm/szab023)

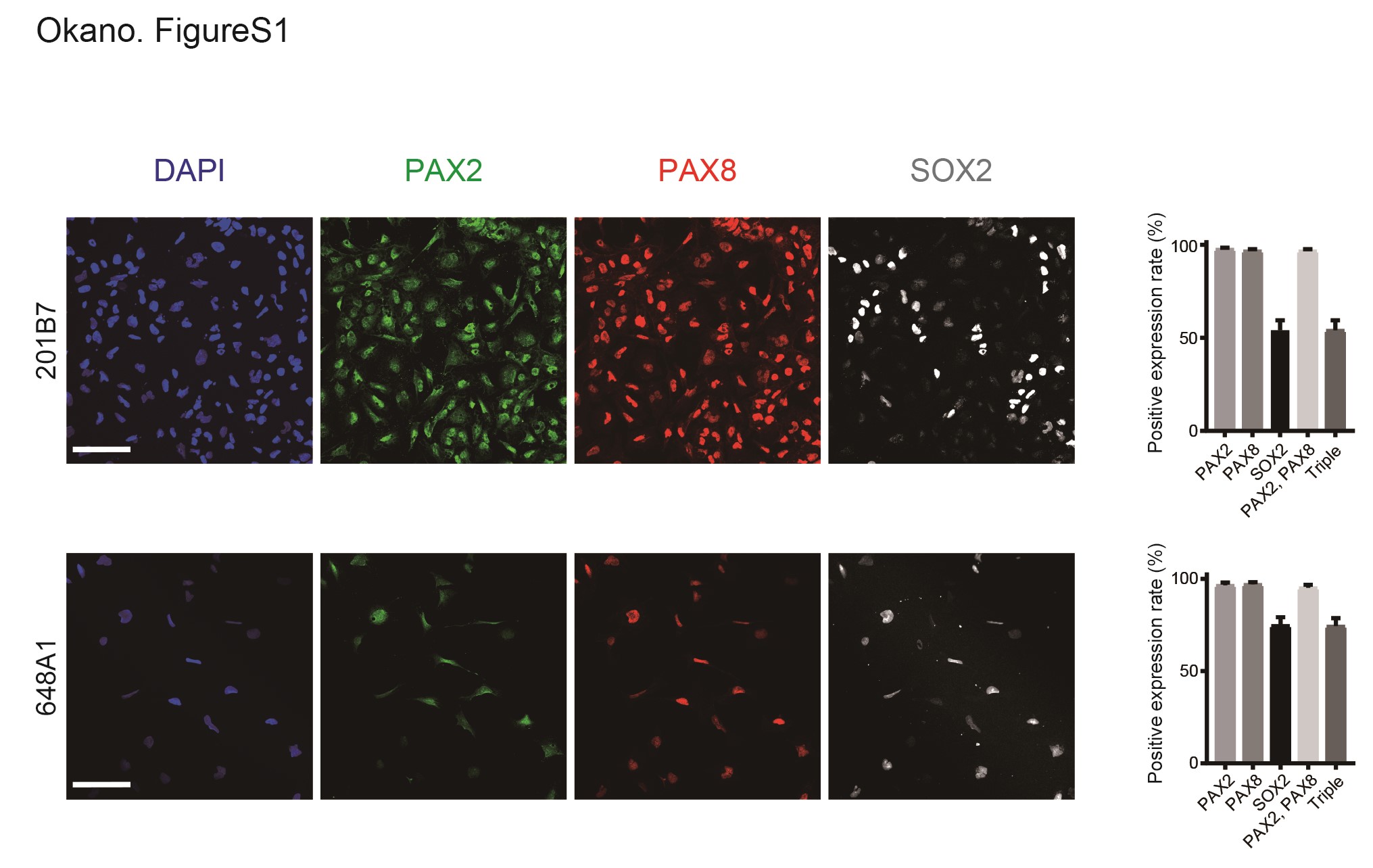

Supplement: szab023_suppl_Supplementary_Figure_S1 [file szab023_suppl_supplementary_figure_s1.jpeg]

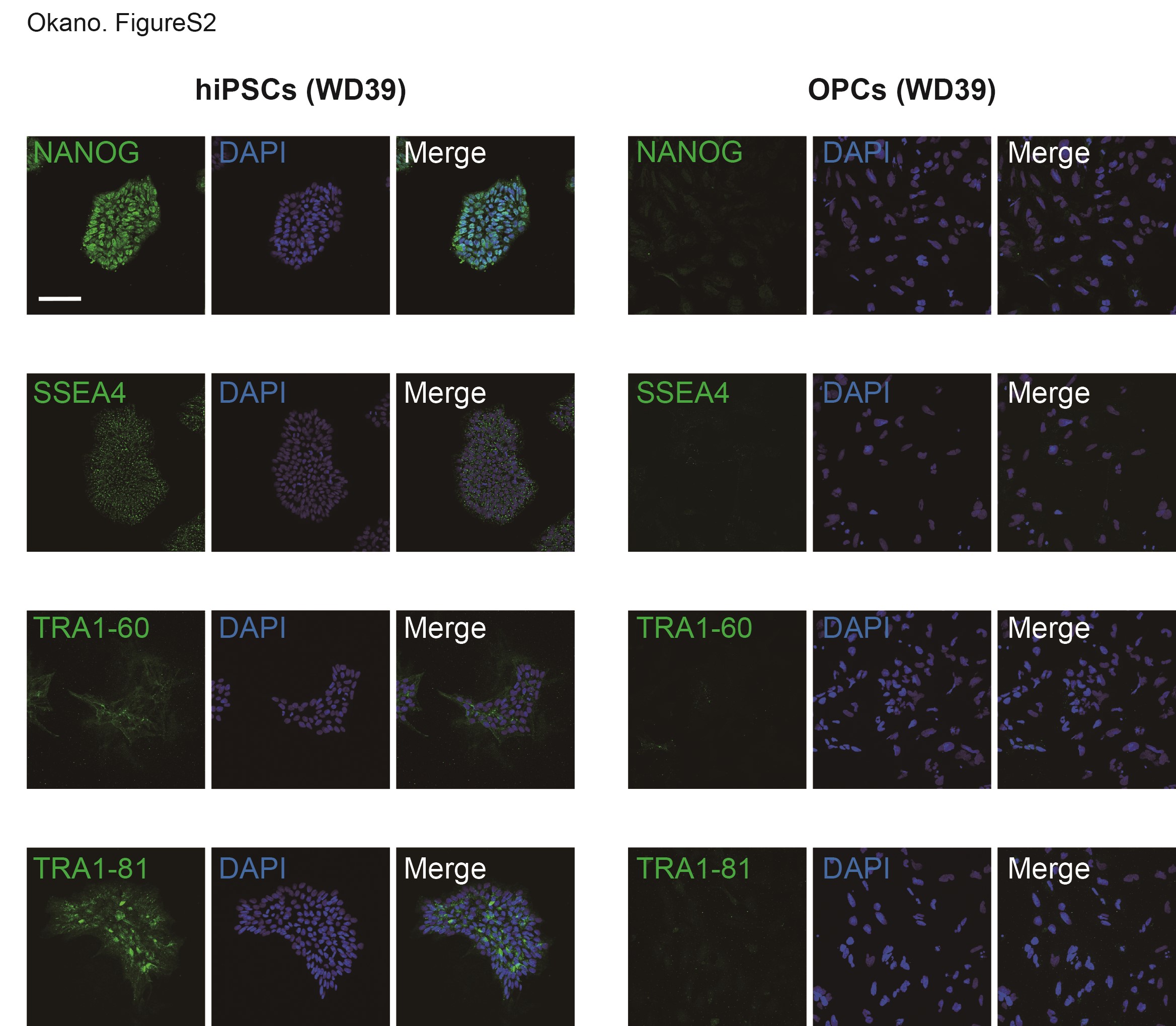

Supplement: szab023_suppl_Supplementary_Figure_S2 [file szab023_suppl_supplementary_figure_s2.jpeg]

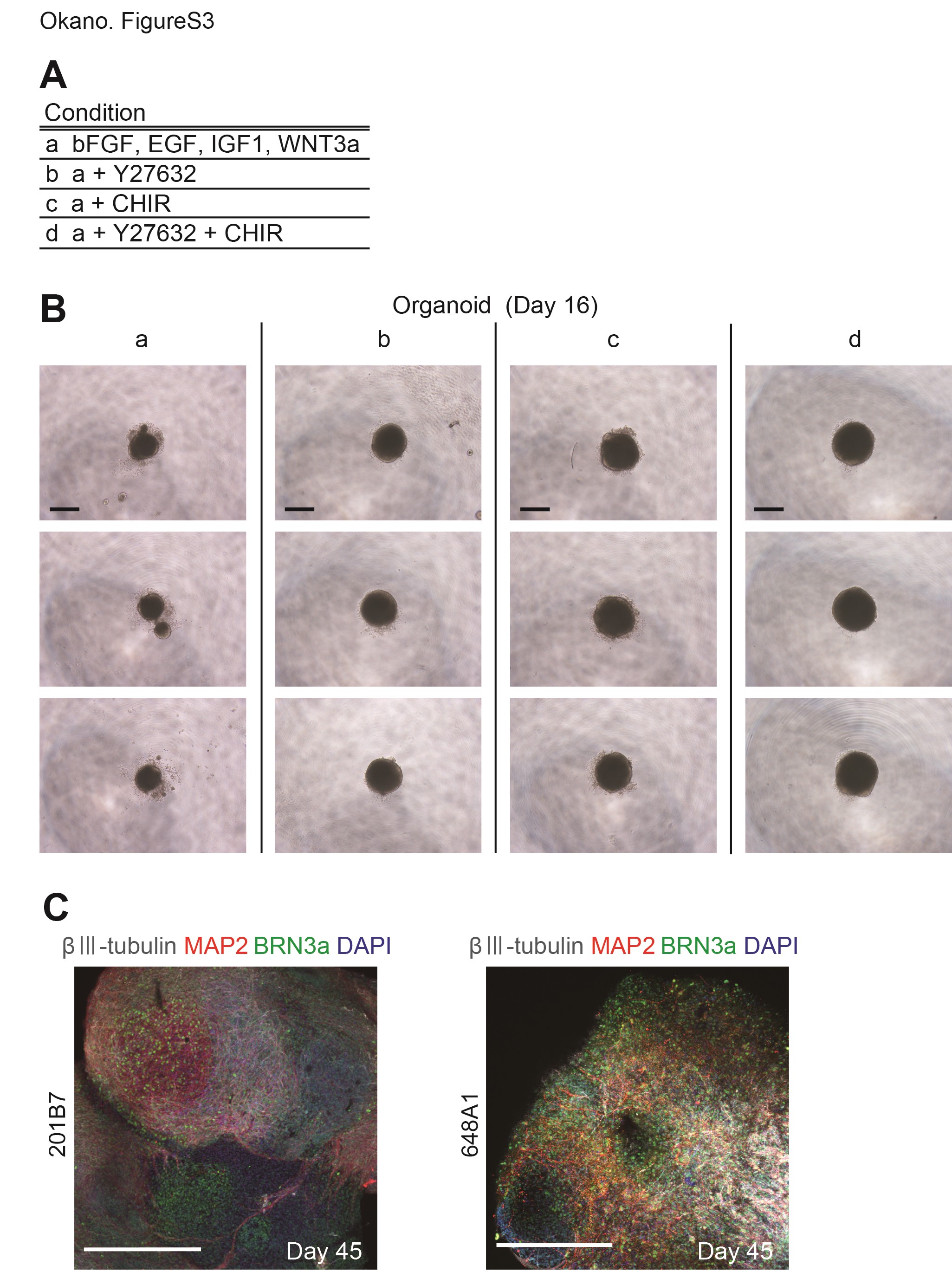

Supplement: szab023_suppl_Supplementary_Figure_S3 [file szab023_suppl_supplementary_figure_s3.jpeg]

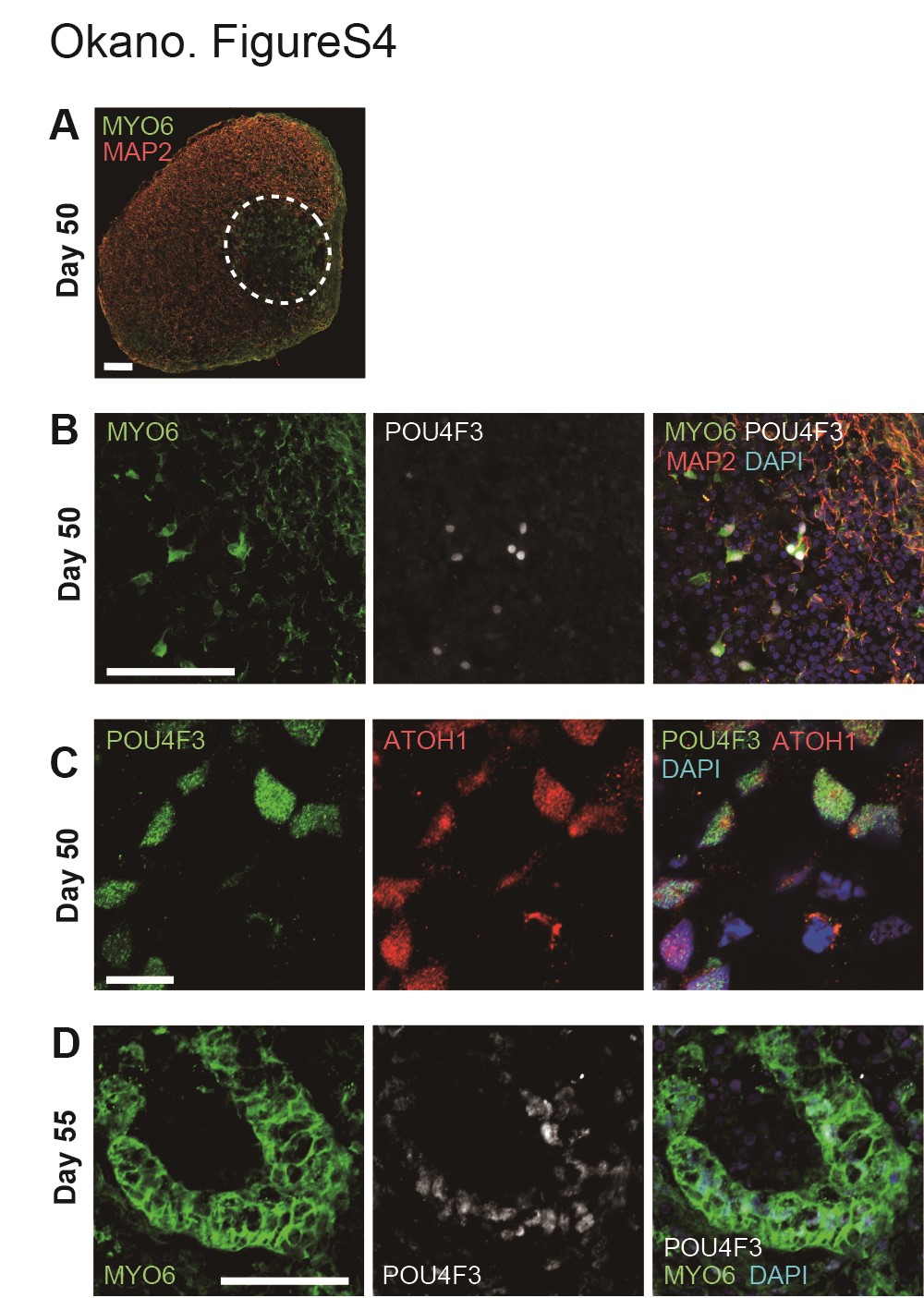

Supplement: szab023_suppl_Supplementary_Figure_S4 [file szab023_suppl_supplementary_figure_s4.jpeg]

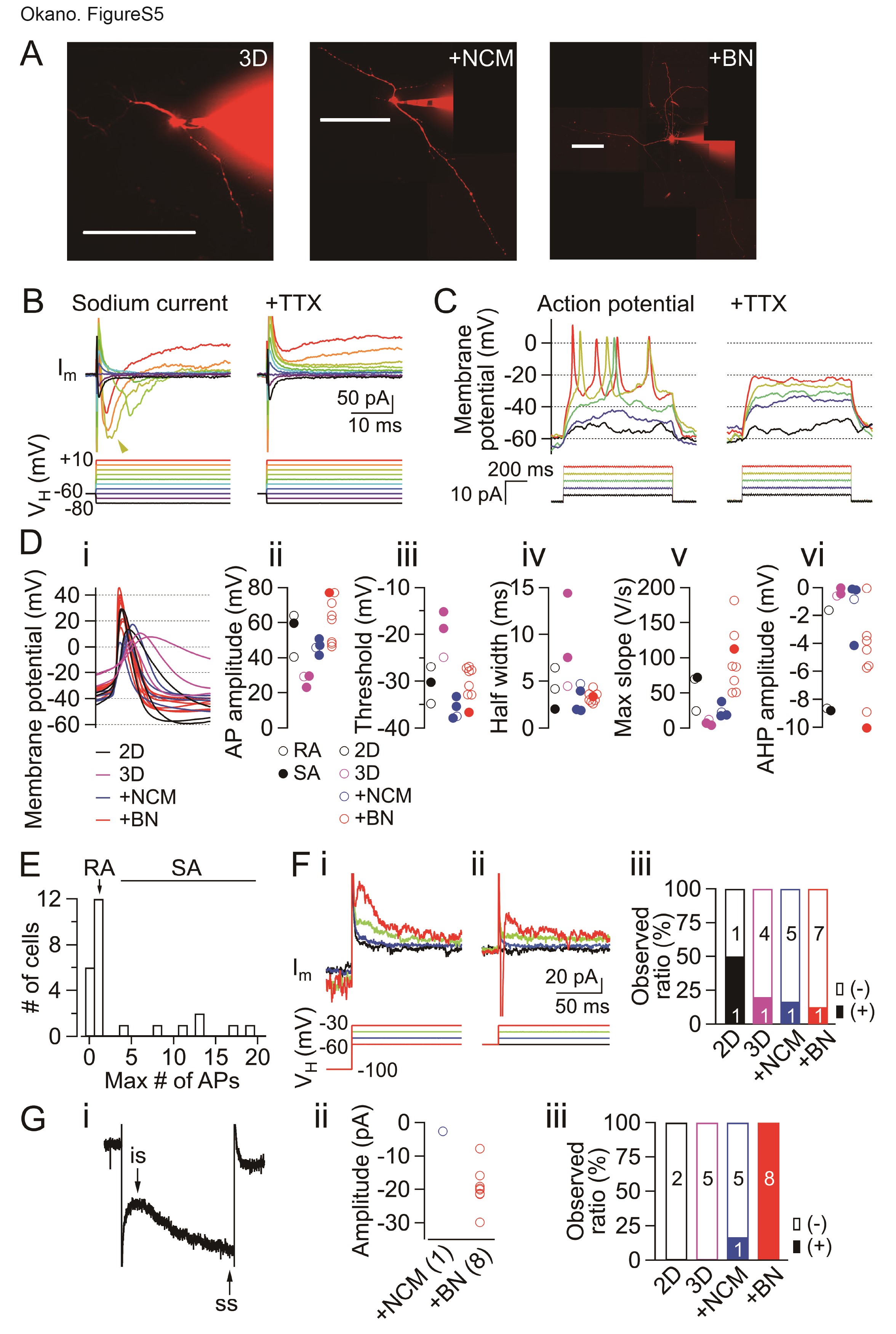

Supplement: szab023_suppl_Supplementary_Figure_S5 [file szab023_suppl_supplementary_figure_s5.jpeg]

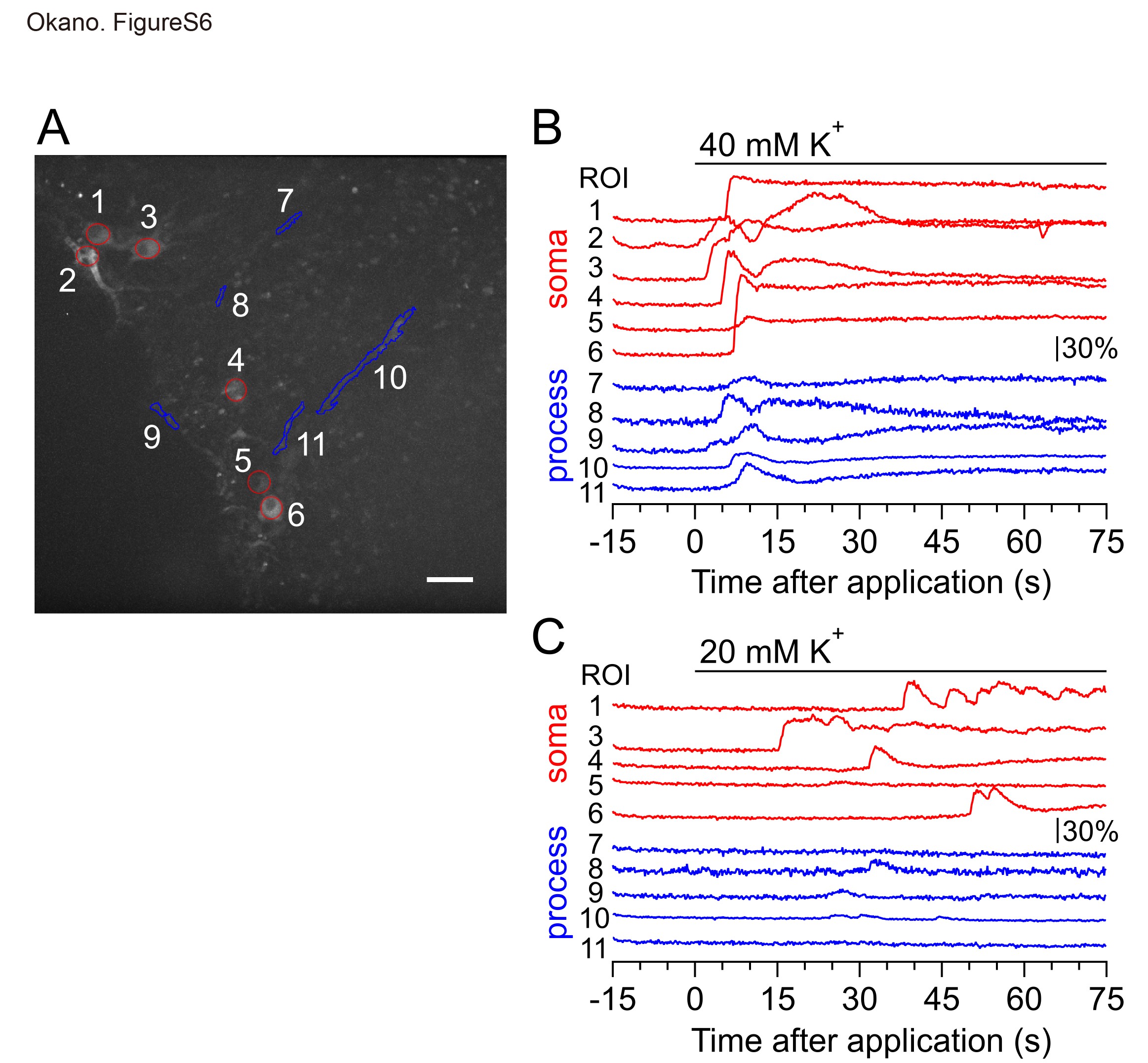

Supplement: szab023_suppl_Supplementary_Figure_S6 [file szab023_suppl_supplementary_figure_s6.jpeg]

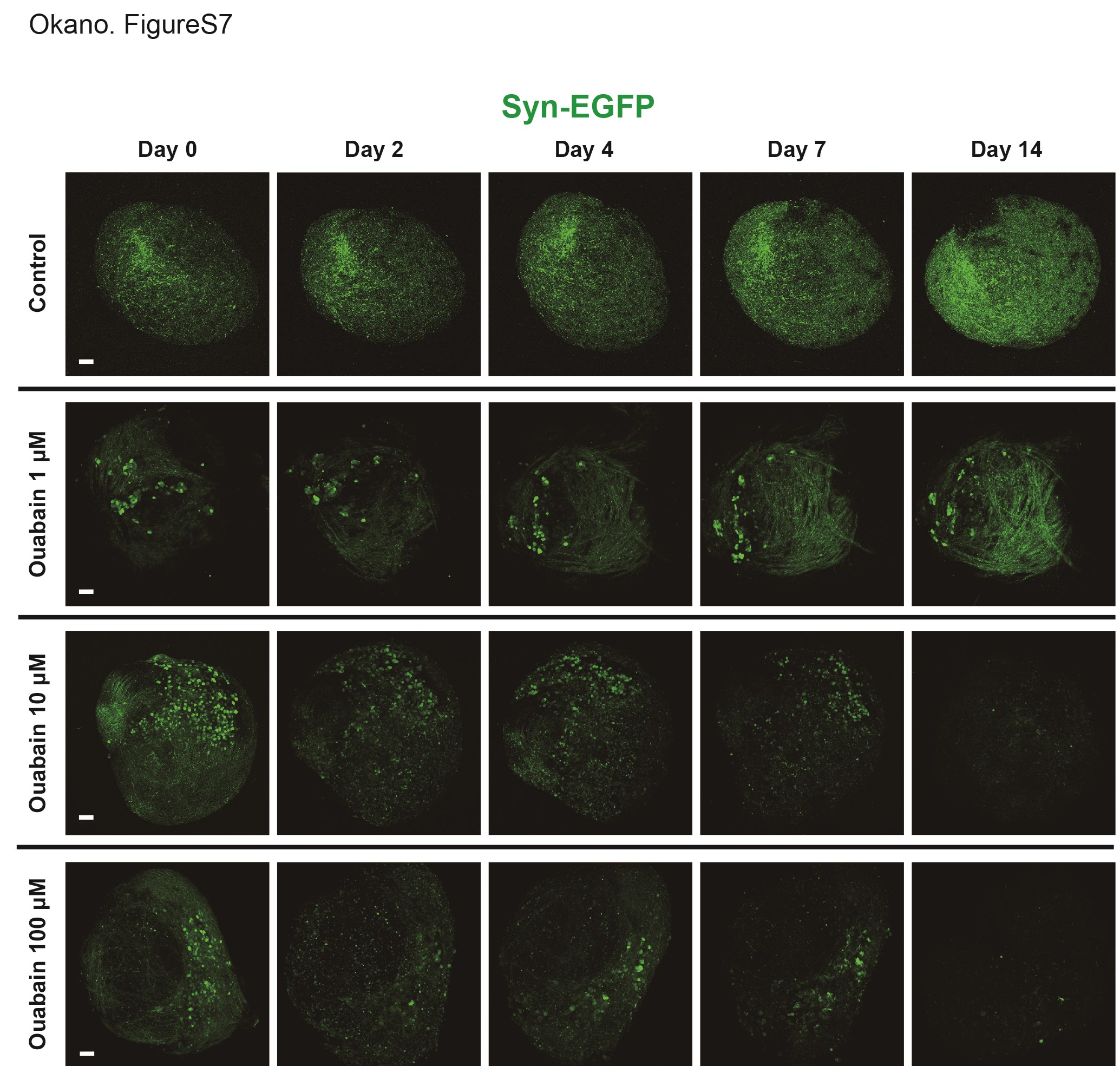

Supplement: szab023_suppl_Supplementary_Figure_S7 [file szab023_suppl_supplementary_figure_s7.jpeg]

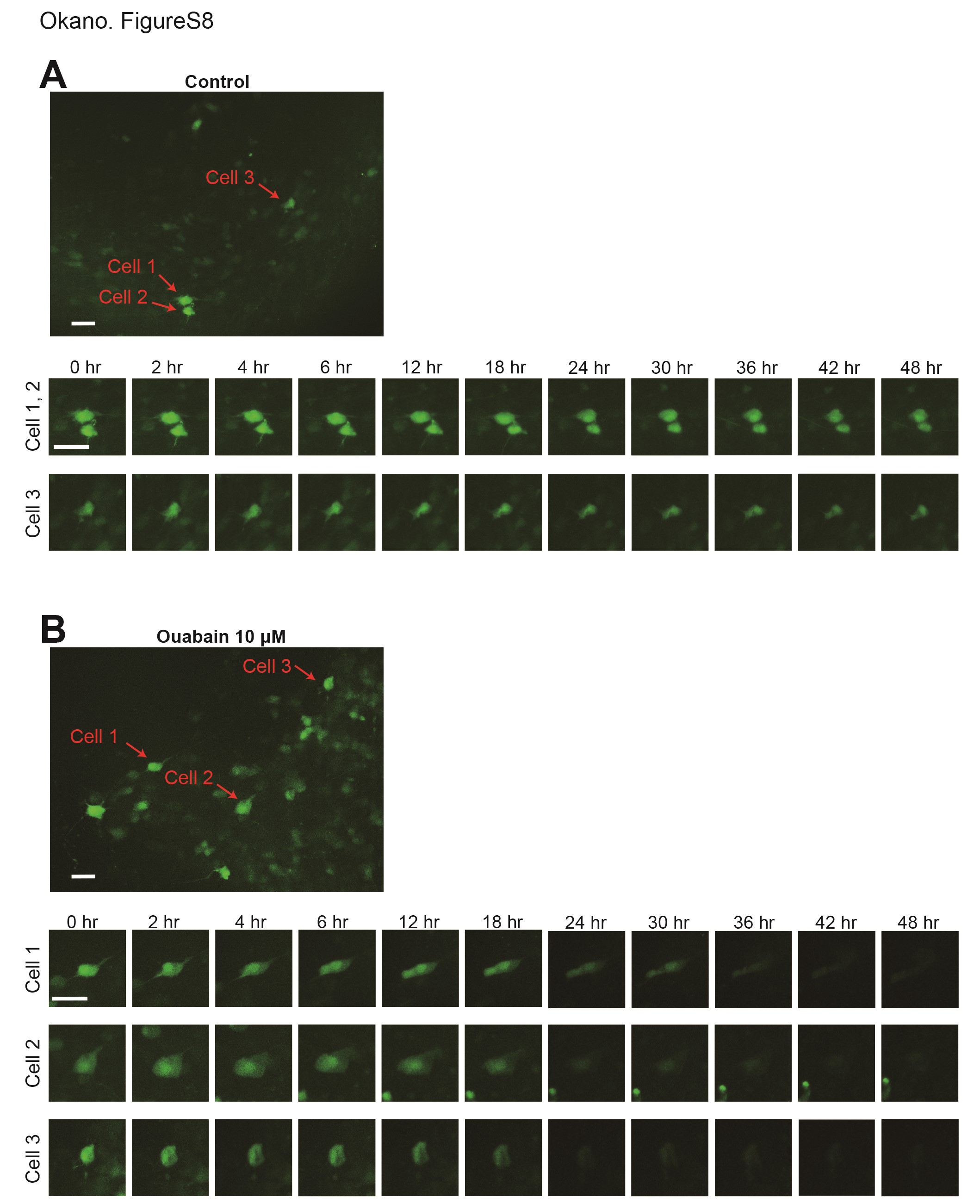

Supplement: szab023_suppl_Supplementary_Figure_S8 [file szab023_suppl_supplementary_figure_s8.jpeg]

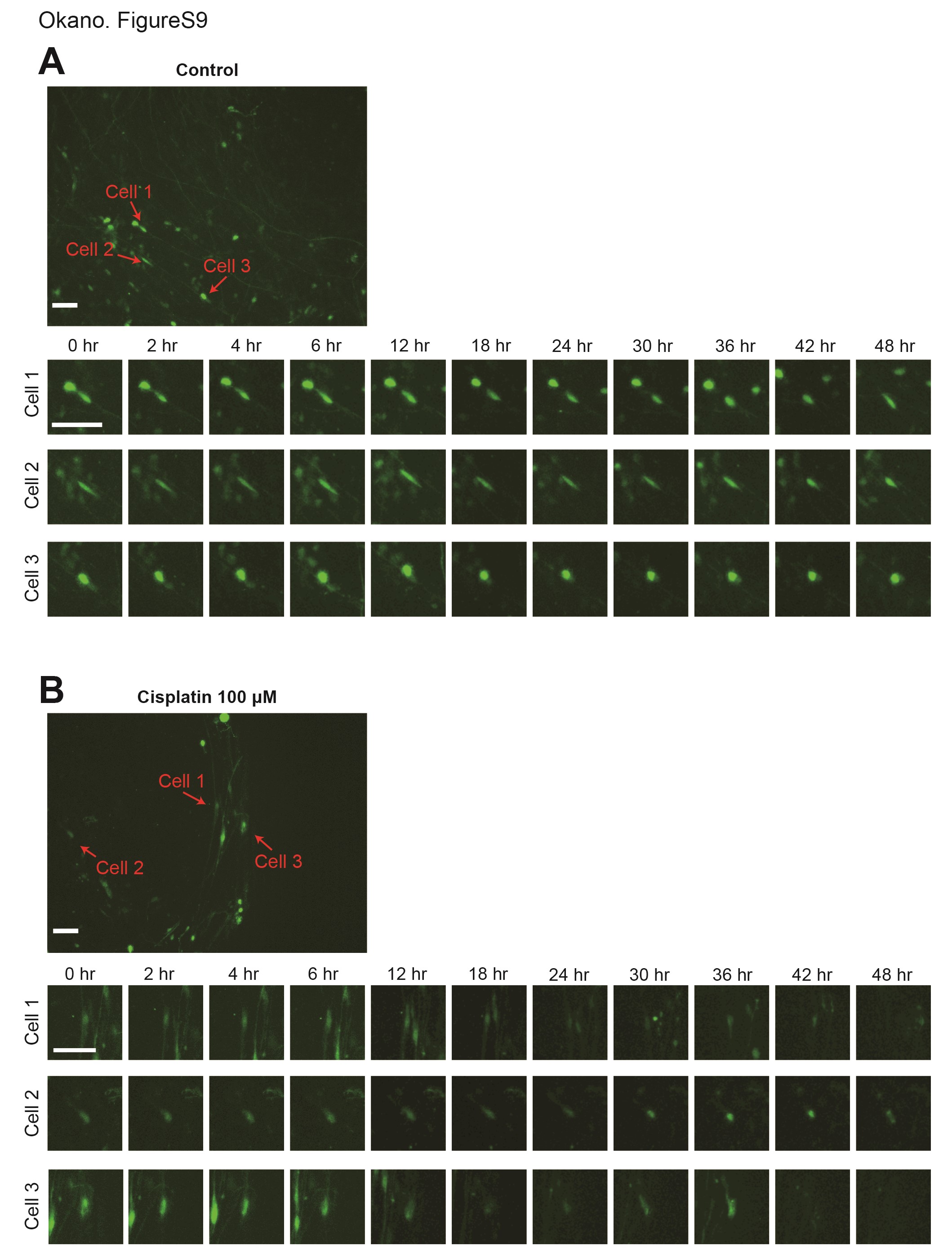

Supplement: szab023_suppl_Supplementary_Figure_S9 [file szab023_suppl_supplementary_figure_s9.jpeg]
