# Supplementary material for: Otic Organoids Containing Spiral Ganglion Neuron-like Cells Derived from Human-induced Pluripotent Stem Cells as a Model of Drug-induced Neuropathy
Source: Stem Cells Transl Med. 2022 Mar 7;11(3):282–96. doi: 10.1093/stcltm/szab023 (PMC8968745; doi:10.1093/stcltm/szab023)
Supplement: szab023_suppl_Supplementary_Tables [file szab023_suppl_supplementary_tables.docx]

**Table S1. Primer sets.**

| Gene | Forward | Reverse |
| --- | --- | --- |
| PAX2 | CAAAGTTCAGCAGCCTTTCC | CCACACCACTCTGGGAATCT |
| PAX8 | TCCAGGAAATCTGGGAAATG | GGGCCCCTTCTATCTGACTC |
| SOX2 | ATGCACCGCTACGACGTGA | CTTTTGCACCCCTCCCATTT |
| GATA3 | TTCTGCCGTACCCAGTTTTT | GGGTCGTTGAATGATTTGCT |
| DLX5 | CCAACCAGCCAGAGAAAGAA | GCAAGGCGAGGTACTGAGTC |
| EYA1 | TTGAAGCCCTGACCGACTC | TTGCTCCTTGTTCTTCTTCTAC |
| FOXG1 | AGAAGAACGGCAAGTACGAGA | TGTTGAGGGACAGATTGTGGC |
| NKX5.1 | GACATGAAGCGCTATCTGAG | AGTTCTCGTGGTAGAGGATG |
| PHOX2B | GTCTGTCTCGGGGAATGTGT | CTGGGTTGTTGGCTTTTTGT |
| ACTIN | GCACCACACCTTCTACAATG | TGCTTGCTGATCCACATGTG |

**Table S2. Dilution of antibodies.**

| Antibody | Dilution |
| --- | --- |
| Rabbit polyclonal anti-Myosin-VI | 1:400 |
| Mouse monoclonal anti-Human beta-Tubulin III | 1:400 |
| Goat polyclonal anti-Sox2 | 1:500 |
| Mouse monoclonal anti-Human PAX2 | 1:200 |
| Rabbit monoclonal Anti-PAX8-C-terminal | 1:400 |
| Mouse monoclonal anti-E-Cadherin | 1:100 |
| Rabbit polyclonal anti-N-Cadherin | 1:400 |
| Rabbit polyclonal anti-Neurogenin 1-N-terminal | 1:400 |
| Mouse monoclonal anti-Human NeuroD1 | 1:400 |
| Mouse monoclonal anti-Gata3 | 1:100 |
| Rat monoclonal anti-GFP | 1:1000 |
| Mouse monoclonal anti-Human Nuclei antibody | 1:100 |
| Mouse monoclonal anti-Nestin | 1:100 |
| Goat polyclonal anti-Jagged1 | 1:100 |
| Chicken polyclonal anti-MAP2 | 1:2000 |
| Mouse monoclonal anti-Brn3c | 1:100 |
| Rabbit polyclonal anti-ATOH1 | 1:400 |
| Rabbit polyclonal anti-Brn3a | 1:400 |
| Guinea pig polyclonal anti-vesicular glutamate transporter 1 | 1:2000 |
| Goat polyclonal anti-Calretinin (Calb2) | 1:200 |
| Rabbit polyclonal anti-Prox1 | 1:200 |
| Mouse monoclonal anti-ATP1A3 | 1:200 |
| Rabbit polyclonal anti-Peripherin | 1:100 |
| Mouse monoclonal anti-Neurofilament H | 1:400 |
| Rabbit monoclonal anti-Cleaved Caspase 3 | 1:200 |
| Mouse monoclonal anti-TRA1-60 | 1:100 |
| Mouse monoclonal anti-TRA1-81 | 1:400 |
| Mouse monoclonal anti-SSEA4 | 1:200 |
| Rabbit polyclonal anti-Nanog | 1:200 |
| Alexa Fluor 405 Donkey Anti-Mouse IgG (H+L) | 1:250 |
| Alexa Fluor 488 Donkey Anti-Mouse IgG (H+L) | 1:500 |
| Alexa Fluor 546 Donkey Anti-Mouse IgG (H+L) | 1:500 |
| Alexa Fluor 647 Donkey Anti-Mouse IgG (H+L) | 1:500 |
| Alexa Fluor 405 Donkey Anti-Rabbit IgG (H+L) | 1:250 |
| Alexa Fluor 555 Donkey Anti-Rabbit IgG (H+L) | 1:500 |
| Alexa Fluor 555 Donkey Anti-Rat IgG (H+L) | 1:500 |
| Cy3-AffiniPure Donkey Anti-Chicken IgY (IgG) (H+L) | 1:500 |
| Alexa Fluor 488 Donkey Anti-Goat IgG (H+L) | 1:500 |
| Alexa Fluor 647 Donkey Anti-Goat IgG (H+L) | 1:500 |
| Alexa Fluor 488 Goat Anti-Mouse IgG (H+L) | 1:500 |
| Alexa Fluor 555 Goat Anti-Rabbit IgG (H+L) | 1:500 |
| Alexa Fluor 647 Goat Anti-Chicken IgY (H+L) | 1:500 |
| Alexa Fluor 555 Goat Anti-Guinea pig IgG (H+L) | 1:500 |
