# Supplementary material for: Otic Organoids Containing Spiral Ganglion Neuron-like Cells Derived from Human-induced Pluripotent Stem Cells as a Model of Drug-induced Neuropathy
Source: Stem Cells Transl Med. 2022 Mar 7;11(3):282–96. doi: 10.1093/stcltm/szab023 (PMC8968745; doi:10.1093/stcltm/szab023)
Supplement: szab023_suppl_Supplementary_Materials [file szab023_suppl_supplementary_materials.docx]

**SUPPLEMENTAL MATERIALS**

**Induction of OPCs**

hiPSCs cultured under feeder-free conditions were dissociated into single cells using Accutase (Innovative Cell Technologies, USA) and passed through a 40-μm cell strainer. The cells were then resuspended in StemFit medium containing 10 mM Y27632 (Wako, Japan) and plated at 10,000–20,000 cells/cm^2^ on iMatrix-511-coated culture dishes. Y27632 was removed from the StemFit medium 24 hours after passaging. Differentiation was initiated (differentiation day 0) by exchanging the medium for serum-free (SF) medium containing 1% N2 supplement (Thermo Fisher Scientific), 2% B27 supplement (Thermo Fisher Scientific), 1× NEAAs, 1× Glutamax (Thermo Fisher Scientific), 0.1 mM 2-mercaptoethanol and 100 mg/mL ampicillin in DMEM/F12. From day 3 to day 5, 25 ng/mL FGF2, 25 ng/mL FGF3 (R&D Systems, USA), 25 ng/mL FGF10 (PeproTech), 25 ng/mL FGF19 (R&D Systems) and 10 ng/mL BMP4 (PeproTech) were added to the SF medium. From day 6 to day 8, 25 ng/mL FGF2, 25 ng/mL FGF3, 25 ng/mL FGF10, 25 ng/mL FGF19 and 8 μM CHIR (Cayman, USA) were added to the SF medium. The medium was changed daily. On day 9, differentiated cells were re-dissociated into single cells using Accutase, resuspended in DMEM/F12 containing 1% N2 supplement, 2% B27 supplement, FGF2, FGF3, FGF10 and FGF19, and passaged on poly-L-ornithine/fibronectin-coated (Sigma-Aldrich) culture dishes. The dishes were then maintained in 5% O_2_. OPCs were obtained the following day.

**Generation of otic spheroids**

Otic spheroids were formed by culturing OPCs under 2D conditions for 2 days and under 3D conditions for 5 days in a hypoxic environment (Figure 3A). OPC medium was exchanged for DMEM/F12 containing 1% N2, 2% B27, 1× Glutamax and 100 mg/mL ampicillin (DFNB medium) and supplemented with 20 ng/mL FGF2, 20 ng/mL EGF (PeproTech) and 50 ng/mL IGF1 (R&D Systems) 24 hours after passaging. The next day, OPCs were dissociated into single cells using Accutase and then resuspended in DFNB medium containing 20 ng/mL FGF2, 20 ng/mL EGF, 50 ng/mL IGF1, 20 ng/mL Wnt3a, 1 μg/mL heparin (R&D Systems), 10 mM Y27632 and 3 μM CHIR. Cell suspensions were transferred into 96-well low-attachment surface U-bottom plates at 1,500 cells per well and maintained in 5% O_2_ for 5 days to generate otic spheroids that formed solid tissues while maintaining progenitor cell traits.

**Generation of otic organoids**

Otic organoids were generated by exposing otic spheroids to 21% O_2_ and removing the mitogenic FGF2 and EGF from the medium at the maturation stage (Figure 3A). On day 16, otic spheroids were transferred to 6-well low-attachment plates at 4 spheroids per well and cultured in 3 mL DFNB medium containing 20 ng/mL IGF1, 10 ng/mL BDNF (PeproTech) and 10 ng/mL NT3 (PeproTech) in 21% O_2_. Half the medium was replaced every 2 days, and organoids cultured for ≥30 days (day 45 from OPC induction) were used for electrophysiological analyses or live-cell imaging.

**RNA isolation and gene expression analysis**

Total RNA was extracted from OPCs on day 10 using TRIzol® reagent (Invitrogen, USA) and an RNeasy® Plus Mini Kit (Qiagen, Germany). First-strand cDNAs were synthesized using ReverTra Ace (Toyobo, Japan) according to the manufacturer’s protocol. PCR amplification was performed using EmeraldAmp enzyme (Takara, Japan). Amplified PCR products were electrophoresed on agarose gels and visualized with ethidium bromide staining. Primer sets are described in Table S1.

For qRT-PCR, TaqMan gene expression assays were performed using predesigned probes (Thermo Fisher Scientific) for the OPC markers, *PAX8* (Hs00247586_m1) and *PAX2* (Hs01057416_m1). qRT-PCR was performed using an ABI 7300 real-time PCR system (Applied Biosystems, USA) and TaqMan Gene Expression Master Mix (Thermo Fisher Scientific). The cDNA content in each sample was calculated using the ΔΔCt method, and target gene expression was normalized to *ACTB* expression (Hs99999903_m1). Experiments were performed in duplicate, and the results are expressed as the mean ± standard error of the mean (SEM).

**Immunofluorescence analyses**

For immunohistochemical analyses, OPCs were fixed with 4% paraformaldehyde for 15 minutes at room temperature, and organoids were fixed overnight at 4ºC. Cryopreserved 10-µm sections were made using a cryostat (HM525, Thermo Fisher Scientific). Cells or sections were incubated in phosphate-buffered saline (PBS) containing 0.3% Triton X-100 for 15 minutes at room temperature. After incubation in blocking buffer (PBS containing 10% goat or donkey serum in 0.1% Triton X-100) for 1 hour at room temperature, cells were incubated with primary antibodies overnight at 4ºC. After three washes with PBS, the cells were incubated with secondary antibodies for 1 hour at room temperature. Nuclei were stained with 1 μg/mL 4′,6-diamidino-2-phenylindole (DAPI; Sigma). Staining was examined using fluorescence microscopy (Zeiss LSM 880 confocal laser-scanning microscope or Olympus IX-77 inverted microscope).

For whole-mount immunostaining, blocking buffer solution (PBS containing 1% bovine serum albumin, 0.3% skim milk and 0.3% Triton X-100) was applied for 1 hour at room temperature before incubation of the specimen with primary and secondary antibodies (24 hours at room temperature on a rotating shaker). Samples were then incubated in ScaleA2 clearing solution (Milli-Q water containing 4 M urea, 10% glycerol and 0.1% Triton X-100) for 4–5 days at room temperature before imaging.^1^ The primary antibody dilutions are described in Table S2.

Semi-automated quantification of cell numbers was performed using IMARIS 8 (Bitplane, UK). The fluorescence intensity of interest (MAP2, TUNEL or GFP) and the background fluorescence intensity were quantified using ImageJ (National Institutes of Health, USA). Fluorescence ratios were obtained by dividing the fluorescence intensity of interest by the background intensity.

For line-scan analysis of MAP2, sections from three otic organoids taken on days 46–50 were stained for MAP2. Four lines (50 μm wide and 500 μm in length) were defined sequentially from the edge of each of the three sections, and the MAP2 fluorescence ratio was calculated using ImageJ. The data were averaged and plotted according to the distance of the line from the edge of the organoid (Figure 4L).

**Staining for dead cells**

TUNEL staining of dead cells in the organoids was performed using a Click-iT TUNEL imaging kit (Thermo Fisher Scientific) just before immunostaining. Organoids were incubated overnight at room temperature for the TdT reaction and for 3 hours at 36ºC for the Click-iT reaction.

Organoids were incubated in DMEM/F-12 (without phenol red) containing 4.5 μM propidium iodide (Sigma-Aldrich) and 1 μg/mL Hoechst 33342 for 15 minutes and then washed with fresh medium. The number of dead cells in each organoid was counted.

**Measurement of ROS production**

Organoids were washed with DMEM/F-12 and incubated first with 5 μM MitoSOX red (Thermo Fisher Scientific) in DMEM/F-12 for 1 hour and then with 1 μg/mL Hoechst 33342 for 15 minutes at 37ºC in 5% CO_2_. Fluorescence images of the organoids were obtained immediately after washing. Since this was an experiment for organoids rather than adherent cells, the incubation time was extended in reference to previous reports.^2^

**2D culture following organoid dissociation**

Organoids at day 20 were dissociated with Neuron Dissociation Solution (Wako, 291-78001) and then plated on Matrigel-coated (Corning, USA) coverslips. Cells were cultured in DFNB medium for 7 days, and the neurites were counted.

**Electrophysiological recordings**

Organoids cultured for more than 40 days were used for patch-clamp recordings. For experiments using the 2D culture condition, organoids were dissociated using Neuron Dissociation Solution and then plated on Matrigel-coated coverslips. Recordings from neurons were obtained after culture in DFNB medium containing IGF1 for 12 hours. For the 3D, +NCM and +BN culture conditions, organoids were exposed to AAV-syn-EGFP 2 weeks before the recordings were made. Organoids were moved to a Transwell insert (Corning, 3401) 12 hours before recording and cultured under air-liquid interface conditions at 37ºC in 5% CO_2_ to fix them to the membrane.

The dispersion cultures or organoids were submerged in solution (125 mM NaCl, 3 mM KCl, 2 mM CaCl_2_, 1.3 mM MgCl_2_, 1.25 mM NaH_2_PO_4_, 10 mM D-glucose, 0.4 mM L-ascorbic acid and 25 mM NaHCO_3_; pH 7.4, gassed with 95% O_2_ and 5% CO_2_; osmolarity, 290–300 mOsm/kg H_2_O) that was continuously superfused at a flow rate of 1–2 mL/min. Membrane currents and potentials were recorded from visually identified bipolar-like cells (2D culture condition) and EGFP-expressing cells (3D, +NCM and +BN culture conditions) with an electron-multiplying charge-coupled device-based Nipkow-disk confocal microscope (iXon Ultra 897, Andor, UK; CSU-X1, Yokogawa Electric, Japan; BX-51WI, Olympus, Japan). An oblique illumination image and a fluorescence image of the same region were obtained, and sequential focal planes separated by 0.4 μm were captured to obtain the projected confocal fluorescence image.

Whole-cell recordings were made using patch-clamp electrodes made from borosilicate glass pipettes (1B120F-4; World Precision Instruments, USA). The composition of the internal solution was: 120 mM potassium gluconate, 6 mM NaCl, 1 mM CaCl_2_, 2 mM MgCl_2_, 2 mM ATP magnesium salt, 0.5 mM guanosine-5'-triphosphate sodium salt, 5 mM ethylene glycol-bis(β-aminoethyl ether)-N,N,N',N'-tetraacetic acid, 12 mM phosphocreatine disodium salt and 10 mM hydroxyethyl piperazineethanesulfonic acid hemisodium (adjusted to pH 7.3 with KOH; osmolarity, 290–300 mOsm/kg H_2_O). Alexa Fluor 568 hydrazide (25 μg/L; Thermo Fisher Scientific) was added to the internal solution when recordings were made from EGFP-expressing cells. The tip resistance of the electrode was 4–7 MΩ. The membrane potential was held at –60 mV during the recordings. The liquid junction potential was not compensated. The membrane capacitance was compensated. All recordings were made at room temperature (20–25ºC) except for recordings of postsynaptic currents, which were made at 30–32ºC to increase the rate of synaptic transmission events. Membrane currents and potentials were recorded with an Axopatch 700B amplifier (Molecular Devices, USA), low-pass filtered at 2 kHz and sampled at 10 kHz with 16-bit resolution (PowerLab, ADInstruments, Australia). The resting membrane potential was measured immediately after rupture of the patch membrane. To record voltage-dependent membrane currents, two voltage step protocols were applied from a holding potential of -60 mV in voltage-clamp recordings: (1) voltage steps (duration of 500 ms) to -20 mV or +10 mV; (2) a fixed hyperpolarizing step to -100 mV (500 ms) followed by voltage steps (500 ms) to a range of potentials from -60 mV to -30 mV in 10 mV increments. In current-clamp recordings, a continuous current was injected manually to keep the membrane potential at approximately -60 mV. Depolarizing step pulses (5 steps with +3, +5, +10, +20 or +30 pA increment; duration of 1 s) delivered 1 s after a fixed-amplitude hyperpolarizing pulse (optimized between -5 to -100 pA to hyperpolarize the cell below -80 mV; duration of 1 s) were applied to identify the firing pattern. The depolarizing protocol was repeated three times for each recording, and the number of overshooting action potentials was counted. Cells showing only a single action potential were defined as rapidly-adapting, and those showing multiple action potentials were defined as slowly-adapting. Tetrodotoxin (1 μM; Alomone, Israel), CNQX (10 μM; Sigma-Aldrich) and D-APV (50 μM; Tocris, UK) were applied in some experiments. The recorded membrane currents and potentials were analyzed off-line with Igor Pro (WaveMetrics, USA).

**Calcium imaging**

Organoids cultured for more than 40 days were used for calcium imaging. Organoids were exposed to AAV-syn-GCaMP6s 2 weeks before the recordings were made. Organoids were placed on Transwell inserts 12 hours before recording and cultured under air-liquid interface conditions at 37ºC in 5% CO_2_ to fix them to the membrane.

Fluorescence images were captured at 6.67 frames/s with a Nipkow disk confocal microscope at an excitation wavelength of 488 nm. Changes in fluorescence from the baseline level (ΔF) were normalized to the average baseline level of fluorescence (F0) before application of a high-potassium solution (the osmolarity of the high-potassium solution was adjusted to 290–300 mOsm/kg H_2_O by reducing the NaCl concentration). The fluorescence images were analyzed using ImageJ.

**Live-cell imaging**

An LSM 880 confocal microscope (Zeiss, Germany) with a ×10 air objective was used for whole-mount live-cell imaging of virally-labeled organoids. A maximum intensity projection image was made from the z-stack images every 24 hours. For live-cell imaging of individual neurons, time-lapse images were captured every hour with a BZ-X700 microscope (Keyence, Japan). At each time point, a z-stack of optically sectioned images was captured and converted to a full-focus image for analysis. Data from three GFP-positive bipolar neurons selected from control and drug-treated organoids were used for the statistical analyses (Figures S8 and S9).

**Virus administration**

AAV5.hSyn.eGFP.WPRE.bGH (titer: 1.42e13 GC/mL; Penn Vector Core, Addgene, USA) and AAV5.Syn.GCaMP6s.WPRE.SV40 (titer: 2.52e13; Penn Vector Core, Addgene) were used for the transduction of genes required for live-cell imaging and calcium imaging, respectively. Viral solution (1 μL) was added to culture medium (100 μL) containing a single organoid. After incubation with virus for 24 hours at 37ºC, the organoid was moved to fresh medium.

**Supplemental reference**

1. Hama H, Kurokawa H, Kawano H et al. Scale: a chemical approach for fluorescence imaging and reconstruction of transparent mouse brain. Nat Neurosci 2011;14(11):1481-1488.
2. Teitz T, Fang J, Goktug AN et al. CDK2 inhibitors as candidate therapeutics for cisplatin- and noise-induced hearing loss. J Exp Med 2018;215(4):1187-1203.
